# DNA-Helix Inspired Wire Routing in Cylindrical Structures and Its Application to Flexible Surgical Devices

**DOI:** 10.1089/soro.2020.0145

**Published:** 2022-04-19

**Authors:** Hwan-Taek Ryu, Se-Min Oh, Kyung Tae, Byung-Ju Yi

**Affiliations:** ^1^ Department of Intelligent Robotic Engineering, Hanyang University, Ansan, Republic of Korea.; ^2^ Department of Dual System Hub Organization, Korea Polytechnic University, Siheung, Republic of Korea.; ^3^ Department of Otolaryngology-Head and Neck Surgery, Hanyang University, Seoul, Republic of Korea.; ^4^ School of Electrical Engineering, Hanyang University, Ansan, Republic of Korea.

**Keywords:** DNA helix, flexible mechanism, laryngopharyngeal surgery, surgical device

## Abstract

In general wire-driven continuum robot mechanisms, the wires are used to control the motion of the devices attached at the distal end. The slack and taut wire is one of the challenging issues to solve in flexible mechanism. This phenomenon becomes worse when the continuum robot is inserted into the natural orifices of the human body, which inherently have uncertain curvilinear geometries consisting of multiple curvatures. Inspired by the unique characteristic of DNA-helix structure that the length of the helix remains almost constant regardless of the deflection of the DNA structure, this article proposes a new idea to design useful flexible mechanism to resolve slack of wires. Using modern Lie-group screw theory, the analytic model for length of helix wire wrapped around a single flexible backbone is proposed and then extended to a general model with multiple flexible backbones and different curvatures. Taking advantage of this helix type wire mechanism, we designed and implemented a flexible surgical device suitable for laryngopharyngeal surgery. The effectiveness of the proposed flexible mechanism is demonstrated through both simulation and phantom experiment.

## Introduction

The biomimetics of DNA characteristic is one of the most interesting issues, and various research fields are inspired by this. In fact, the helix structure of the DNA inspires the antenna related field to increase the bandwidth^1,2^ and optical related field as broadband circular polarizers using square arrays of three-dimensional (3D) gold helices^3–5^ and parallel double helix wire-shaped supercapacitor.^6^ Moreover, the helix structure is applied to the actuator materials inspired by the biomimetic helical fiber topologies for artificial muscles,^7^ in the construction of tubular electromechanical actuators based on polypyrrole,^8^ and superhydrophobic helix to transport the bubble in an aqueous environment.^9^

With respect to the DNA nanotechnology, the DNA structure has inspired the biological ion channels,^10,11^ and stress sensitive hydrogel was controlled using the characteristic of DNA nanoswitch.^12^ Moreover, design of biomimetic catalyst inspired by the double-helix morphology is proposed,^13^ and DNA biomimetic constructions are applied to mimic the physical properties and functions of lipid membranes.^14^

### Related researches: application of helix structure to continuum mechanism

The helix structure has inspired various components that make up the continuum mechanism. With respect to the helical wire routing, wire with helix of large pitch was arranged to miniaturize routing of cables^15^ and to make more dexterous motion in distal end of steerable part^16^ or its spiral configuration.^17,18^ It was also applied to helical arrangement of fibers to generate the wormlike motion (axial extension, radial expansion).^19^ Furthermore, the statics and dynamics of continuum robot considering straight and helical wire routing and distributed loads have been analyzed in depth,^20^ and spiral insertion technique for endoscope was studied.^21^

### Wire slack problems on surgical device and existing methods to resolve

**FIG. 1.**
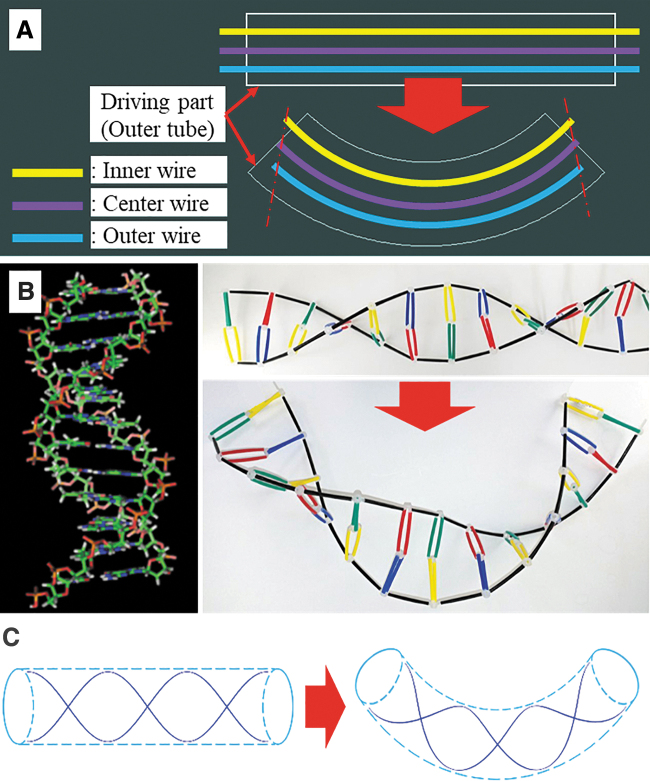
Proposal of DNA-helix inspired flexible mechanisms. **(A)** The problem related to the variation in the lengths of wire when the flexible cylinder is deflected. The wire slackness at the inner part and the wire tautness at the outer part occur. **(B)** The structure of the Nucleic acid double helix and its simplified representation. It is observed that there is no length change in double stranded DNA helix upon deflection. **(C)** The proposal of the flexible cylinder structure, including the wire arrangement in helix pattern. The flexible mechanism is inspired by the constant length of DNA helix. Color images are available online.

As reported in many research articles, there are many shortcomings of wire-driven systems used in minimally invasive surgery.^22,23^ They include irregular deflection of continuum bodies inside the human body, length changes, and wire slack and hysteresis problems when controlling the wires. The problem related to the variation in the lengths of wire is explained in Figure 1A, where three wires penetrate into a flexible cylinder and their role is to control any device at the distal end of the flexible cylinder by pulling wires. As shown in Figure 1A, the length of lumen inside the flexible cylinder varies their lengths once the cylinder is bent. The inner lumen will be shortened, and the outer lumen will be extended.

As a result, wire slackness occurs at the inner part and wire tautness occurs at the outer part. Such length variation of lumen causes wire slackness or disconnection, causing latency in the motion response or payload shortage during operation of end tools such as grippers, biopsy devices, and electrocauteries. Therefore, design of the wire transmission line is an important issue, which has been considered as a serious problem to solve in many engineering fields. There have been several methods to compensate for wire slack as follows:

(1) Mechanical method: application of tension to the wire using a spring or elastic elements,^24–26^
(2) Structural method: joint pulley method to compensate the changes in inner and outer wire length or change the wire direction,^27–30^
(3) Algorithmic method: compensation or prediction of the length of the wire using the algorithm.^31^

The method using elasticity does not work when the pulling force caused by the tautness of the wire is more than the linear range of the elastic force. That is, the elastic force is reduced every time under repeated bending. In structural method, the diameter of the joint pulley has a great effect. The continuum robot mechanism based on the joint pulley can be also used in a narrow and crooked environment, but it is prone to be stuck in the environment. Moreover, using additional mechanical parts is still its shortcoming. In the proposed method, we do not use additional components. For algorithmic method, the solution to compensate the length change in driving part is task specific according to the characteristics of each system. So there is no general method. Moreover, its performance is deteriorated when the continuum robot consists of several curvature sections, and there exists unexpected curvature in the system. As a result, the existing solutions are not appropriate to design of flexible surgical device to resolve the wire slack problem in narrow workspace without using additional components.

### Proposal

DNA structure can be recognized as a virtual cylinder with two helices wrapped around its surface. Several acids connecting two helices enable the whole structure to behave like a flexible cylinder. This structure maintains the characteristics of the cylinder even after bending the entire structure (Fig. 1B). However, there is no prior work using such a DNA structure to design robotic devices such as surgical tools, endoscopes, and catheter. Considering the structural characteristics of DNA, the wire slack problems occurring in engineering problems can be resolved by introducing the idea of DNA helix that maintains its length even upon deflection of the DNA structure. In this article, a continuum model with double and multiple helix strand structure will be examined along with their applications (Fig. 1C).

First of all, the analytic model for length of the helix is driven for a single flexible backbone with multiple sections using the Lie-group modern screw theory. To remove any slack or shortage of wire upon deflection of mechanism, a flexible mechanism is designed such that multiple wires pass through the hollow helix lines or multiple wires are wrapped around a flexible backbone with a period of 2π. Then, devices located at the distal end of the corresponding flexible devices or systems can be controlled properly without suffering any wire slack or disconnection due to the length change in wire lumen.

Initially, we prove this phenomenon through the fundamental experiment and then demonstrate the effectiveness of DNA helix wire through design and experiment of a flexible endoscope suitable for laryngopharyngeal surgery. It is expected that the results of this work can be applied to many applications requiring wire-driven principle without having slack of wires.

## Material and Method

### Kinematic modeling of the helix wires around the curved backbone

As one of the contributions of this article, we propose the kinematic modeling of the helix wires around the curved backbone using modern Lie-group screw theory. The typical relative transformation method based on Denavit–Hartenburg parameters can be also used in the kinematic modeling instead. However, the Lie-group screw theory is effective with respect to its simple representation, and it is also computationally cheap using less number of products in transformation.

Consider a flexible cylinder and helix wires wrapped around the cylinder. The center line of the cylinder is called the backbone. Suppose that the flexible cylinder and its backbone form an arc with respect to the instantaneous center of rotation and the modeling of this flexible rod and helix wire is premised on Kirchhoff's rod theory, which states that the centerline of an elastic rod is inextensible and whose cross sections remain plane and normal to the centerline.^32–34^ This assumption may hold when the diameter or the dimension size of the cross-sectional area is much smaller than the length of the centerline of the cylinder. The Young's and shear moduli of the cylinder are also considered to be constant.

**FIG. 2.**
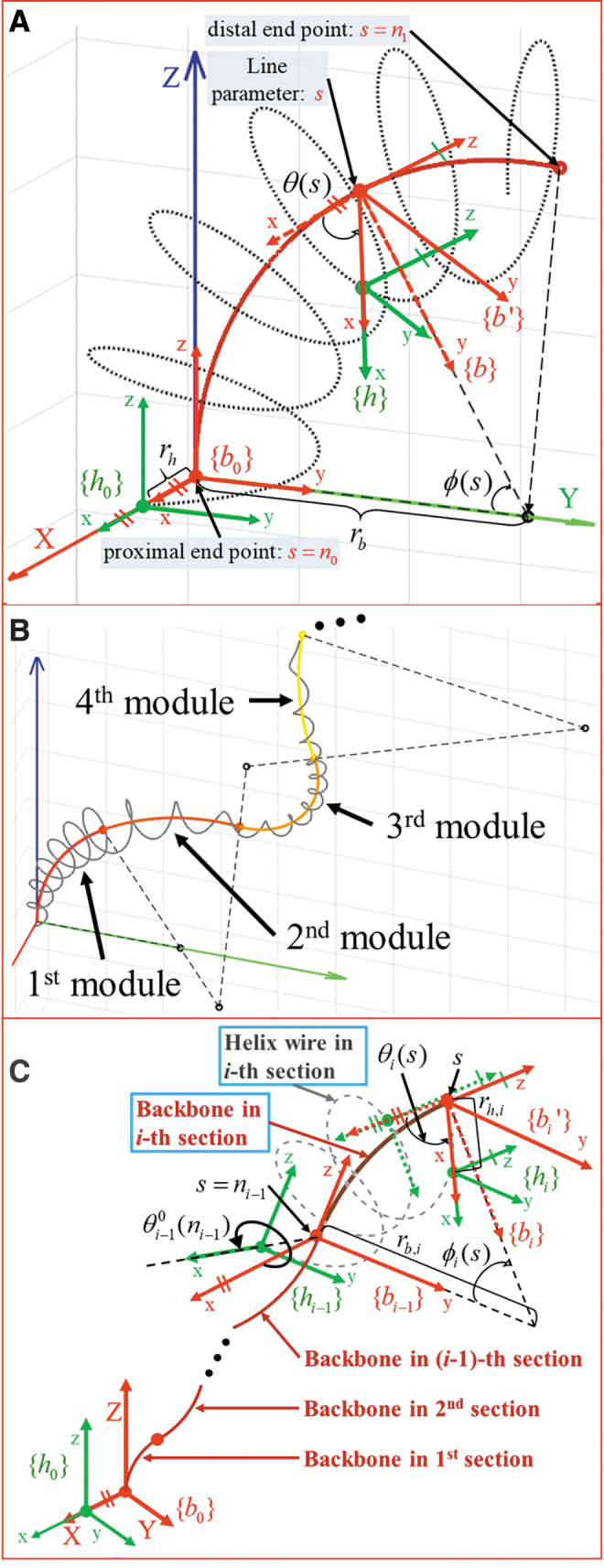
Modeling of the backbone curves and the helix wires. **(A)** Coordinate frames of the curved backbone rod and helix wire wrapped around the backbone rod for one module analysis. **(B)** Concept of combination of the backbone curves and the helix wire modules for general modeling. **(C)** Kinematic information of backbone and helix modeling in *i*-th module is presented as an example. Color images are available online.

In this subsection, the position and orientation of the curved backbone and the helix wire are solved with respect to the length parameter *s* for one module with a specific radius of curvature. The coordinate frames of the curved backbone and helix wrapped around the flexible cylinder are described in Figure 2A, where the red line denotes the backbone configuration with radius of curvature *r_b_*.

Assuming that the cross-sectional area of the flexible body maintains a circle even upon deflection for low curvature and constant curvature, the radius of the helix is denoted as *r_h_*. *n*_0_ and $n_{1}$ denote the proximal and distal end point of the curved backbone, respectively. $\left\{b_{0}\right\}$ and {b} denote the coordinate of the backbone located at $s=n_{0}$ and any $s\in \left[n_{0},n_{1}\right]$, respectively. The local *z*-axis of the backbone frame {b} is set tangential to the backbone curve. Similarly, $\left\{h_{0}\right\}$ and {h} denote the frames of helix wire located at $s=n_{0}$ and any $s\in \left[n_{0},n_{1}\right]$, respectively. The origin of the helix frame {h} is at the point where the plane perpendicular to the tangent of the backbone curve at *s* intersects with the helix wire.

ϕ(s) denotes the bending angle of the backbone, which is measured from the *y*-axis of the backbone frame $\left\{b_{0}\right\}$ to the *y*-axis of the backbone frame {b} about the global *x*-axis located at the center of the curvature. θ(s) denotes the angle of the helix turn at *s*, which is measured from the *x*-axis of the backbone frame {b} to the *x*-axis of the intermediate backbone frame {b′}. As the first step, the transformation of the helix frame {h} relative to $\left\{b_{0}\right\}$ can be solved as the exponential coordinate given by Ref.^35^

(1) ghb0(ϕ(s),θ(s))=eξ^bϕ(s)eξ^hθ(s)ghb0(ϕ(n0),θ(n0))=10000cosϕ(s)sinϕ(s)rb(1−cosϕ(s))0−sinϕ(s)cosϕ(s)rb sinϕ(s)0001⋅…cosθ(s)−sinθ(s)00sinθ(s)cosθ(s)0000100001⋅100rh010000100001=Rhb0(s)p_hb0(s)0_1,

where $R_{h}^{b0}\left(s\right)$ and ${\underline{p}}_{h}^{b0}\left(s\right)$ denoting the rotation matrix and the position vector of the helix frame {h} are as follows:

$$R_{h}^{b0}\left(s\right)=\left[\begin{array}{ccc} \cos\theta \left(s\right) & -\sin\theta \left(s\right) & 0\\ \cos\phi \left(s\right)\sin\theta \left(s\right) & \cos\phi \left(s\right)\cos\theta \left(s\right) & \sin\phi \left(s\right)\\ -\sin\phi \left(s\right)\sin\theta \left(s\right) & -\sin\phi \left(s\right)\cos\theta \left(s\right) & \cos\phi \left(s\right) \end{array}\right]$$

and

p_hb0(s)=rh cosθ(s)rh cosϕ(s)sinθ(s)+rb(1−cosϕ(s))−rh sinϕ(s)sinθ(s)+rb sinϕ(s).

It is noted that the bending angle is given as 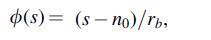, and the helix turn angle is denoted as 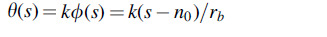 on the premise of the constant pitch of helix. *k* denotes the ratio between θ and ϕ. Then, $R_{h}^{b0}\left(s\right)$ and ${\underline{p}}_{h}^{b0}\left(s\right)$ of Equation (1) are rewritten with respect to the length parameter *s* as

(2) $$\begin{array}{cc} & R_{h}^{b0}\left(s\right)=\left[\begin{array}{ccc} c\left(ks_{r}\right) & -s\left(ks_{r}\right) & 0\\ c\left(s_{r}\right)s\left(ks_{r}\right) & c\left(s_{r}\right)c\left(ks_{r}\right) & s\left(s_{r}\right)\\ -s\left(s_{r}\right)s\left(ks_{r}\right) & -s\left(s_{r}\right)c\left(ks_{r}\right) & c\left(s_{r}\right) \end{array}\right],\\ & {\underline{p}}_{h}^{b0}\left(s\right)=\left[\begin{array}{c} r_{h}c\left(ks_{r}\right)\\ r_{h}c\left(s_{r}\right)s\left(ks_{r}\right)+r_{b}\left(1-c\left(s_{r}\right)\right)\\ -r_{h}s\left(s_{r}\right)s\left(ks_{r}\right)+r_{b}s\left(s_{r}\right) \end{array}\right], \end{array}$$

where 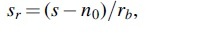, c(⋅) and s(⋅) in Equation (2) are cos(⋅) and sin(⋅), respectively. Finally, the length of the helix wire is calculated using the line integral along the line parameter *s* as follows.

(3) Lh(s)=∫n0sdp_hb0(s)dsTdp_hb0(s)dsds=∫n0srhrb sinksr−12+krhrb2ds

Equation (3) is used to estimate the length of helix wire either when they are wrapped helically around a flexible backbone or they pass through the hollow helix line inside a flexible mechanism.

The generalized modeling is demanding when dealing with flexible cylinders used for general 3D environment. As the second step, we consider flexible cylinder composed of multisections such as colonoscope and endoscope, which usually form multiple curvatures inside the human body. Thus, modeling of a helix wire surrounding an arbitrarily curved backbone is feasible by separating the backbone into *i*-th sections, each of which has a constant curvature as described in Figure 2B. The coordinate frames of the curved backbone and helix wrapped around the flexible cylinder for *i*-th section are described as Figure 2C.

$r_{b,i}$, $r_{h,i}$, $\phi_{i}\left(s\right)$, and $\theta_{i}\left(s\right)$ denote the radius of the curved backbone and helix, bending angle, and helix turn in *i*-th section, respectively. $\left\{b_{i}\right\}$ and $\left\{h_{i}\right\}$ denote coordinates of the backbone and helix wire for *i*-th section located at line parameter *s*. Applying the modeling for the one module case to *i*-th case, the transformation of the helix frame $\left\{h_{i}\right\}$ relative to $\left\{b_{0}\right\}$ can be solved as the exponential coordinate given by

(4) $$\begin{array}{cc} & g_{hi}^{b0}\left(\phi_{i}^{}\left(s\right),\theta_{i}^{0}\left(s\right)\right)=g_{b\left(i-1\right)}^{b0}\left(\phi_{i-1}\left(n_{i-1}\right)\right)\cdot g_{hi}^{b\left(i-1\right)}\left(\phi_{i}\left(s\right),\theta_{i}^{0}\left(s\right)\right)\\ & =\left(\prod_{j=1}^{i-1}e^{{\hat{\xi }}_{bj}\phi_{j}\left(n_{j}\right)}\right)\left(e^{{\hat{\xi }}_{bi}\phi_{i}\left(s\right)}\cdot e^{{\hat{\xi }}_{hi}\theta_{i}^{0}\left(s\right)}\cdot g_{hi}^{b\left(i-1\right)}\left(\phi_{i}\left(n_{i-1}\right),\theta_{i}^{0}\left(n_{i-1}\right)\right)\right).\\ & =\left[\begin{array}{cc} R_{hi}^{b0}\left(s\right) & {\underline{p}}_{hi}^{b0}\left(s\right)\\ \underline{0} & 1 \end{array}\right], \end{array}$$

where $\theta_{i}^{0}\left(s\right)=\theta_{1}^{}\left(n_{1}\right)+\theta_{2}^{}\left(n_{2}\right)+\ldots +\theta_{i-1}^{}\left(n_{i-1}\right)+\theta_{i}^{}\left(s\right)$ denotes the helix turn in *i*-th section, being accumulated from the frame $\left\{h_{0}\right\}$. It can be also expressed as $\theta_{i}^{0}\left(s\right)=\theta_{i-1}^{0}\left(n_{i-1}\right)+\theta_{i}^{}\left(s\right)$. Similar to Equation (3), the length of the total helix wire is calculated by summation of the length of helix wire of all sections as follows.

(5) $$L_{h}\left(s\right)=\sum_{j=1}^{i-1}L_{h,j}\left(n_{j}\right)+L_{h,i}\left(s\right)$$

where

Lh,i(s)=∫ni−1sdp_hib0(s)dsTdp_hib0(s)dsds=∫ni−1sdp_hib(i−1)(s)dsTdp_hib(i−1)(s)dsds=∫ni−1srh,irb,i sinkisrb,i−12+kirh,irb,i2ds

denotes the length of helix wire for *i*-th section, and *k_i_* denotes the ratio between $\theta_{i}\left(s\right)$ and $\phi_{i}\left(s\right)$. Equation (5) is used to estimate the length of helix wire either when they are wrapped helically around a flexible backbone or they pass through the hollow helix line (i.e., lumen) inside a flexible mechanism.

## Results

**FIG. 3.**
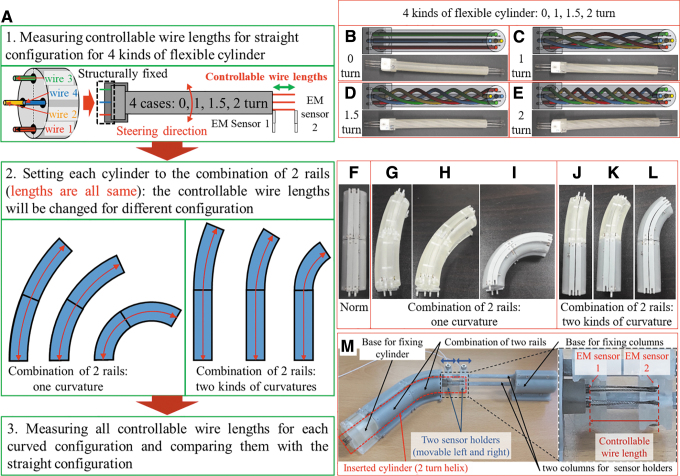
Environment of fundamental experiment. **(A)** Basic concept and procedure of the fundamental experiment. Four kinds of flexible cylinders and its design for **(B)** 0 turn helix, **(C)** 1 turn helix, **(D)** 1.5 turn helix, and **(E)** 2 turn helix. Four flexible cylinders will be inserted into seven kinds of rails. The guide rail has two patterns. The first one has two sections with same curvature **(F)** infinite curvature for straight rail, **(G)** 200 (mm) curvature, **(H)** 100 (mm) curvature, **(I)** 50 (mm) curvature. The second one has two sections with different curvatures **(J)** infinite and 200 (mm), **(K)** infinite and 100 (mm), **(L)** infinite and 50 (mm). **(M)** The environmental setting for fundamental experiment. Color images are available online.

The purpose of fundamental experiment is to identify the length variation of wire lumen when the configuration of the flexible body is changed. The specific process of the fundamental experiment and its details are described in Figure 3A.

As the first step, four kinds of the flexible cylinders (0 turn, 1 turn, 1.5 turn, and 2 turn helix wire arrangement) are constructed as Figure 3B–E, respectively. In this study, *N* turn helix wire implies that the helix wire is turned around the backbone by 2πN radian. When we define the number of helix turn, it is based on the certain length of backbone. In the design of the flexible cylinder, the length of backbone is 100 (mm) for which the number of helix will vary from 0 turn to *N* turn. The 0 turn helix pattern implies that the wires only pass through the straight lumens. The specific design is shown as Supplementary Figure S1 in “Construction of the Flexible Cylinder in Fundamental Experiment” section and in Supplementary Table S1. The flexible cylinders were fabricated using 3D printing technology (refer the Supplementary Table S2 for details of 3D printer and materials). In this experiment, four wires are used, since the medical flexible devices such as colonoscope and endoscope are usually driven by four wires to control two-degree of freedom (DOF) steering motion at the distal end. It is also possible to arrange 6, 8, and more wires to produce more DOFs at the distal end. The proximal end of each wire is fixed to one end of the cylinder, but the distal end of the wire is set free.

As the second step, the guide rails were designed to set the configuration of the flexible cylinder. Using four kinds of rail element (radius of curvature: infinity, 200, 100, and 50 (mm). Arc length of each rail element is 50 (mm)), seven kinds of rail configuration were selected in this experiment (Fig. 3F–L). Flexible cylinders with different helix patterns will be inserted into seven kinds of the rail configuration.

As the third step, the flexible cylinder is set to the straight rail (Fig. 3F). Then the length of each controllable wire is measured, and it is used as the standard value. Changing the rail configuration to other six kinds of configuration (Fig. 3G–L), the variation of each controllable wire length is compared with the standard one. To measure the controllable wire length, two electromagnetic (EM) sensors are used. To verify the effectiveness of the proposed models, the measured controllable wire lengths are converted to the length of wire lumen in flexible cylinder and then compared with helix length of simulation results using Equations (3) and (5).

**FIG. 4.**
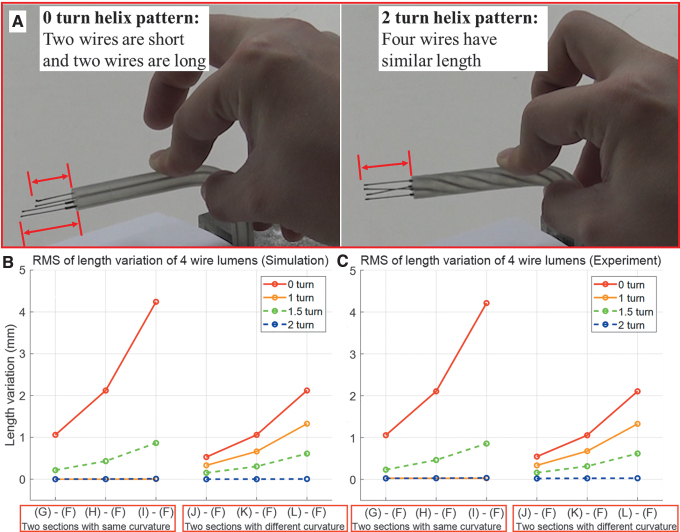
Results of fundamental experiment for difference of controllable wire length. **(A)** Demonstration of the performance for wire arranged in helix between 0 turn and 2 turn helix. It is shown that the controllable wire length is not changed for flexible cylinder, which has 2 turn helix pattern although it is curved. The tendencies of RMS for length difference of four wire lumens in **(B)** simulation and **(C)** experiment are presented. RMS, root mean square. Color images are available online.

Demonstration of performance for variation of the controllable wire length between 0 turn and 2 turn is presented in Supplementary Movie S1, and its snapshots are presented in Figure 4A. It is obviously shown that the two controllable wires become longer and the other two wires become shorter for 0 turn case when the flexible cylinder is curved. In contrast, it is shown that the lengths of four controllable wires maintain constant for 2 turn case even though the flexible cylinder is curved.

The results of the fundamental experiment for simulation and experiment are presented in Figure 4B and C, respectively. The experiment was conducted 20 times. The abscissa implies the case of comparison. For instance, “(G - F)” denotes the difference of the length variation of wire lumens when the flexible cylinder is inserted into the guide rail configuration of Figure 3G and F, respectively. The notations from (H - F) to (L - F) in Figure 4 follow the same manner. The values used in ordinate of Figure 4B and C denote root mean square (RMS) for length difference of four wire lumens in simulation and experiment, respectively. For four kinds of helix turn of wire lumen in flexible cylinder and six kinds of different guide rail setting, they were calculated as follows:

(6) $$\begin{array}{cc} & RMSforsimulation=\sqrt{\frac{1}{n}\sum_{i=1}^{n}L_{i}^{2}}\\ & RMSforexperiment=\sqrt{\frac{1}{n\cdot o}\sum_{j=1}^{o}\sum_{i=1}^{n}L_{i,j}^{2}} \end{array}$$

where nando denote the number of wire lumen for one kind of cylinder and repetition number of experiment, respectively. Its specific values are presented as Supplementary Table S3.

In Supplementary Table S3, the difference of RMS for the length variation of wire lumen between simulation and experiment was found from −3.095 · 10^−2^ to 2.934 · 10^−2^ (mm), which is negligible for all cases. Therefore, effectiveness of modeling solving the length of wire lumen is verified. As a common trend observed in all cases, the more the cylinder is curved, the more the RMS of length variation for four wire lumens is changed. The 0 turn cylinder, which has been used in general continuum devices, has the largest change in length of wire lumen. This phenomenon causes slack of the wire, resulting in delay of motion or loss of payload when pulling wires. It is noted from the experimental data that the 1 turn helix shows considerable effectiveness in preventing wire slack for one curvature environment (Fig. 3G–I), and the 2 turn helix shows considerable effectiveness in all six cases (from Fig. 3G–L). This is because such cases exhibit little length change in the wire lumen although the configuration of the flexible body is changed. This phenomenon is due to a counter-balancing effect occurring when the helix wire lumen turns the outer curve and the inner curve in turn.

In contrast, considering the two kinds of curvature (Fig. 3J–L) and 1 turn helix cylinder cases, the length differences in wire lumen are observed. This is because the 1 turn helix cylinder passes through one half straight lumen and another half curved lumen. There is no length variation for each wire lumen in the half straight section. However, there is no counterbalancing effect in the other half curved section with the curvature since the wires in this section only have a half turn of the helix by π radian. Based on the same principle, the 1.5 turn helix from Figure 3G–L also is not recommended for the counterbalancing effect. However, if the 1.5 turn helix is set to an environment with a constant curvature during the 2/3 of the cylinder length and then a straight section during last 1/3 of the cylinder length, there will be no length variation for the four wires. Summarizing the results mentioned above, the properties of the helix wire arrangement are inferred as follows:

(1) If the backbone has constant curvature during each pitch of the helix wire, there is no length change for the controllable wire because of the counterbalancing effect on the wire turn. On the contrary, if the curvature varies during each pitch of the helix, length variation of the controllable wire occurs.
(2) It is advisable to set the number of helix turns as a positive integer number to obtain the counterbalancing effect, assuming that the cylinder is set to a constant curvature environment.
(3) The more turns in a helix wire for the same cylinder length, the less length variation for the controllable wire length, since it causes a smaller helix pitch and less curvature variation during each helix pitch section. However, too many turns cause an increase in friction force because the cylinder includes a longer length of helix wire and contact area. Thus there should be some compromise in deciding the number of helix turns.

## Application

### Motivation to a surgical device for laryngopharyngeal surgery

**FIG. 5.**
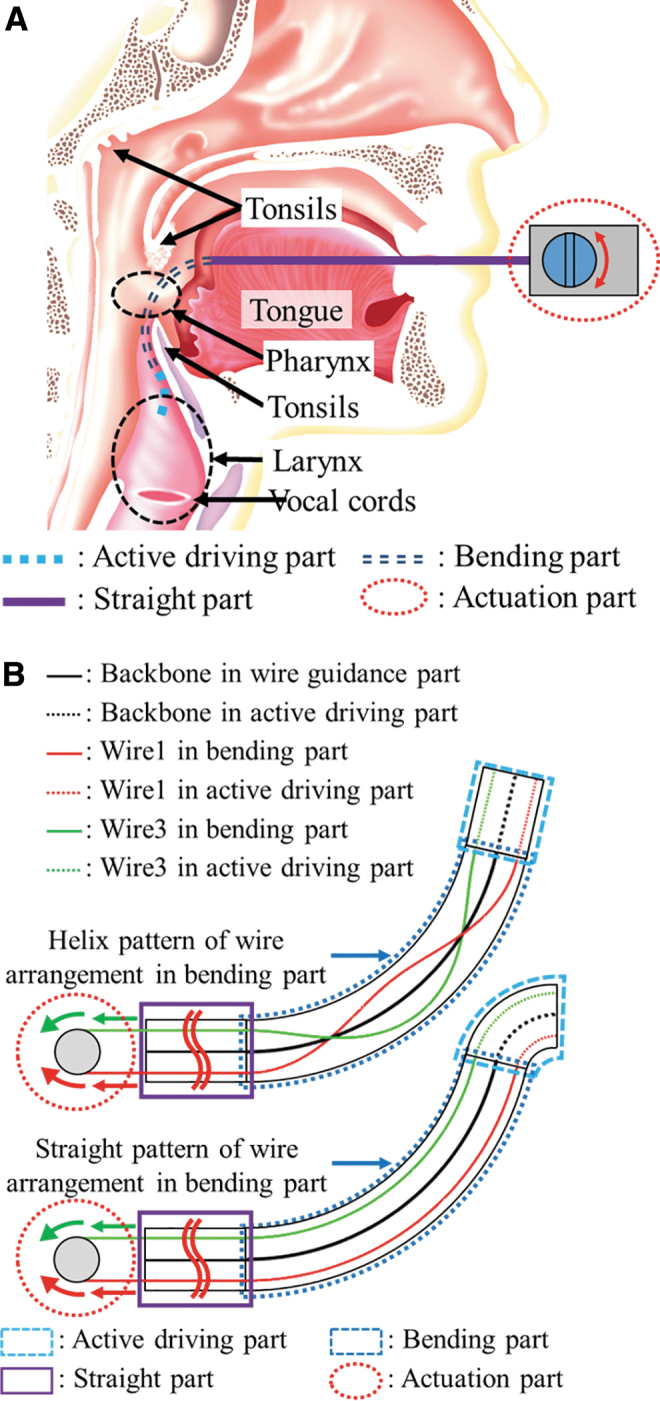
The concept of DNA-helix inspired flexible mechanisms. **(A)** Anatomical structure around the larynx and pharynx and workspace for laryngopharyngeal surgery. Because of its crooked environment, introduction of the helix pattern in wire arrangement greatly enhances the performance of the surgical tool. **(B)** The difference between helix wire and no helix wire arrangement in the bending part. When using the straight pattern of wire in the bending part, the active driving part is bent with some curvature in opposite direction to the curvature of bending part to compensate the wire slack and strain problem in the active driving part. Color images are available online.

In laryngopharyngeal surgery treating lesions in pharynx, larynx, tonsils, and vocal cords, the pathway of the air (namely, workspace) consists of a straight area, bending area, and working area around the target lesion (Fig. 5A). The workspace of laryngopharyngeal surgery is so small (i.e., 30 × 30 × 30 (mm)). So the size of the surgical device should be compact. So using other methods employing large device or additional components to resolve the wire slack problem is not appropriate to this application. This is the main reason that we chose laryngopharyngeal surgery as an example. The functional requirement for a surgical manipulator for laryngopharyngeal surgery is to provide bilateral motion for cautery and gripper without suffering any motion delay and loss of payload due to slack of wire.

There has been a couple of transoral robotic surgery (TORS). The da Vinci surgical robot has been previously used for TORS. However, it is too big to be used for TORS, and especially, it is not adequate for hypopharyngeal lesions located deep in the throat. The FLEX robotic system of Medrobotics was developed exclusively for laryngopharyngeal surgery.^36^ A steering mechanism used in FLEX is a multijoint flexure mechanism that enters the curvature of the throat. This mechanism is driven by the traction of multiple wires driven by motors from the outside, controlling the direction of the foremost node to treat the lesion at the larynx. The backbone at the center of the mechanism follows behind it, creating a mechanical fixation and bend. A camera was attached at the distal end of the mechanism to provide images of the larynx. Two guide tubes are mounted on both sides of the mechanism, through which surgeons insert a passive end effector directly. However, since the curvature of the larynx varies from patient to patient and the path to the larynx can be composed of multiple curvatures, the wire lumen may not be constant in length. It may cause intrinsic wire slack problems, resulting in malfunctioning of the end effectors. To solve this difficulty basically, the DNA helix inspired flexible mechanism is applied to the laryngopharyngeal surgery.

### Construction of surgical device

According to the structure of the pathway, the structure of the surgical device is designed to have four parts: actuation part (handle part), straight part, bending part, and active driving part with surgical tool. In this study, the section with helix wire will be called as the wire guidance part. The actuation part is a handle operated by the surgeon in a passive manner instead of telerobotic system.^37^ The active driving part is to create the steering motion of the end effector at the distal end. Figure 5B demonstrates two different cases of wire arrangement: helix-wire and straight-wire pattern. In case of using helix-wire pattern, the configuration of the active driving part is not affected by the curvature of the bending part. This is because there is no change of wire lumen length in bending part due to using helix type wire arrangement. However, in case of using straight pattern, the configuration of the active driving part is considerably affected by the curvature of the wire guidance part. This is because the wire slack or strain problem occurs at the bending part when it is bent.

As a result, the active driving part will bend with some curvature in the direction opposite to the bending part to compensate the wire slack and strain problem in the bending part. The resulting curvature of the wire guidance part may cause irregular deformation of the active driving part, which is not helpful in operation of the surgical tool located at the distal end of the endoscope. We demonstrated this phenomenon more clearly in Supplementary Data through 3D simulation of the two cases. Conclusively, introduction of the helix pattern in wire arrangement greatly enhances the performance of the surgical tool since it is not affected by the bending of the surgical tool.

**FIG. 6.**
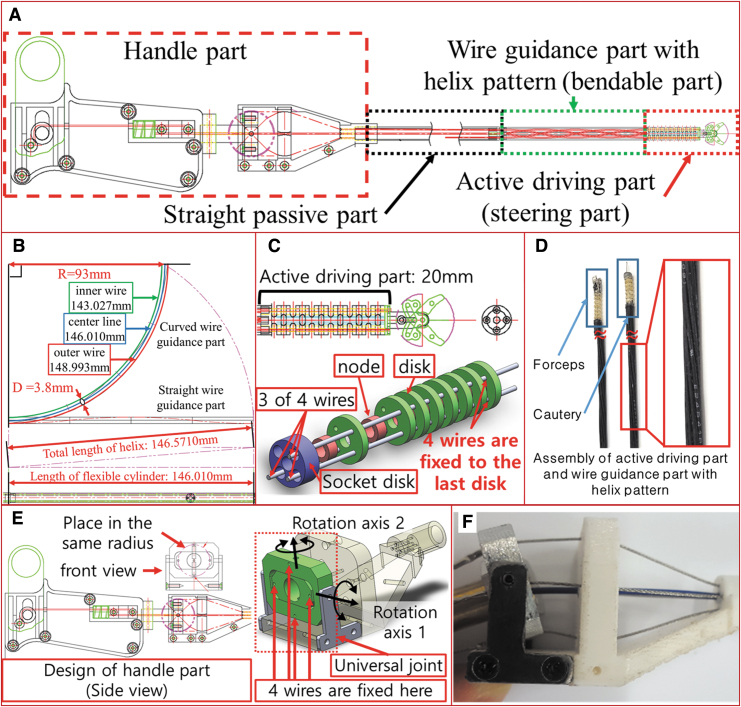
Construction of surgical device for the laryngopharyngeal surgery. **(A)** The whole design of surgical device for the laryngopharyngeal surgery. **(B)** Analysis for length of wire lumen with straight and helix arrangement. **(C)** Specific design concept of active driving part: combination of disks and nodes. **(D)** Two kinds of prototypes for assembly of active driving part and wire guidance part. It is noted for active driving part that four wires are arranged in straight pattern to directly transmit power by pulling wires, and four wires are connected to the distal end of the active driving part. **(E)** Specific design concept of the handle part. A universal joint was used to pull four wires along two rotational axes. **(F)** Prototype of the handle part. Tilting motion of handle part along the rotation axis 1 is demonstrated. Color images are available online.

Adapting the DNA-helix wire concept, the whole design of surgical device for the laryngopharyngeal surgery is proposed as Figure 6A. Figure 6B shows the design of wire guidance part for straight and curved configurations. The length of the wire guidance part is 146.010 (mm), and the diameter of the wire arrangement is 3.8 (mm). Then, length of the wire lumen is also 146.010 (mm). As an example, the radius of curvature is given as 93 (mm), and the flexible device is bent by 90 (deg). For the case of straight arrangement of wire lumen, the length difference between the center lumen and the inner or outer lumens is calculated as 2.983 (mm), and its error rate is 2.04% of the whole length of wire lumen. Such a difference in length of wire lumen would cause problems in control of the end tools due to wire slack.

To resolve such a problem, the flexible cylinder using helix type wire arrangement was designed. Even if the bending part is bent by 90 (deg), the length of each wire lumen arranged with helix is 146.5711 (mm) according to the calculation in Equation (5), which is almost the same as length of wire lumen for straight configuration of wire guidance part (i.e., 146.5710 (mm)). The error rate for length of the wire lumen is 0.000068%, which shows the effectiveness of the proposed helix type wire. The curvature of this bending part will depend on the anatomical structure of each patient. However, the curvature of the throat varies from patient to patient. Thus, to maintain a constant length of wire lumen in the bending part regardless of the curvature, employing helix pattern in wire arrangement is very useful.

Considering operation in the working area (Fig. 5A), the active driving part was designed as Figure 6C. The active driving part of the surgical device consists of a multisectional flexible part and a tool such as a gripper or an abrasion device. The multisectional flexible part is assembled by seven modules, each of which consists of one node and one disk. Each disk has four holes near the surface of the disk for each wire path. Four wires passing through the four holes in the disk are fixed to the distal disk to make steering motion in active driving part caused by pulling wire. The socket joint is connected to the wire guidance part. It is noted that the four wires are arranged in a straight pattern to create the steering motion, but the helix pattern in wire arrangement is only applied to wire guidance part. Since the active driving part usually requires two steering DOF, four wires are used to realize such motions. One set of wires (i.e., two wires) will control each steering angle.

Analyzing the helical type wire arrangement in bending part (Fig. 6B) and considering the working area and performance for active driving part (Fig. 6C), prototype of the wire guidance part and the active driving part is designed as Figure 6D. Considering the operation for laryngopharyngeal surgery, two kinds of end tools such as surgical forceps and cautery are attached on the distal end of the active driving part.

The handle mechanism of continuum device is used to pull wires that are connected to the steering part of the surgical device more efficiently. Many researches related to the passive-type continuum mechanism have proposed the handle mechanism congruent as the steering part.^16,38,39^ However, it means that the effort for construction and maintenance of continuum device becomes twice because of its similar structure between steering part and handle part.

In this article, the handle mechanism using one universal joint is proposed for easier construction and maintenance of the handle mechanism. The specific design and prototype of the handle mechanism is presented as Figure 6E and F, respectively. As shown in Figure 6E and F, two of four wires are fixed to the first joint of the universal joint, and the other two wires are fixed to the second joint of the universal joint to pull one pair of wire with one joint motion. It is noted that four holes to fix each wire in handle part are placed with the same radius at the intersection point of two axes of universal joint.

**FIG. 7.**
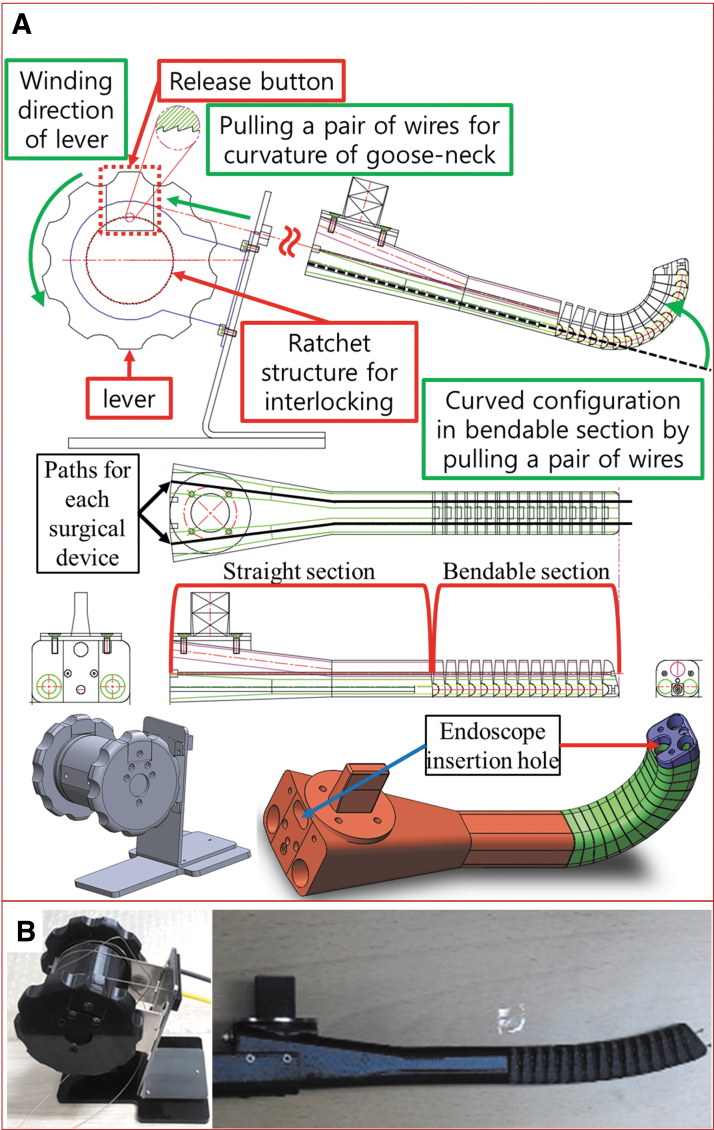
Construction of gooseneck mechanism for the laryngopharyngeal surgery. **(A)** Design concept of gooseneck mechanism, including lever and ratchet structure, to control the curvature of the bendable section in gooseneck mechanism. **(B)** Prototype of the gooseneck mechanism. Color images are available online.

To apply this surgical device for laryngeal surgery, an outer tube should be used, which can be either flexible or rigid. It plays the role of guide rail for the wire guidance part of the surgical device. For this purpose, a gooseneck mechanism was designed (Fig. 7A) and constructed as prototype (Fig. 7B). The curvature of gooseneck device is controlled by pulling or releasing a pair of wires, and winding or release of a pair of wires is controlled by rotating the lever. The desired curvature of gooseneck can be set by interlocking of ratchet structure, and it can be released as unlocking of ratchet structure by pushing the release button. The specifications of the experimental environment for the surgical device, including gooseneck, are presented as Supplementary Table S4.

### Demonstration

As the first step, the motion of the surgical device is tested by manually operating the handle part for various curvatures of the bending part (refer to “Free motion in the air” in Supplementary Movie S2). As the next step, a gooseneck device was used to guide the motion of the surgical device. It was observed that the surgical devices can be operated properly without any motion delay regardless of the curvature angle for both steps. This implies that there is negligible wire slack due to helix arrangement in wire.

**FIG. 8.**
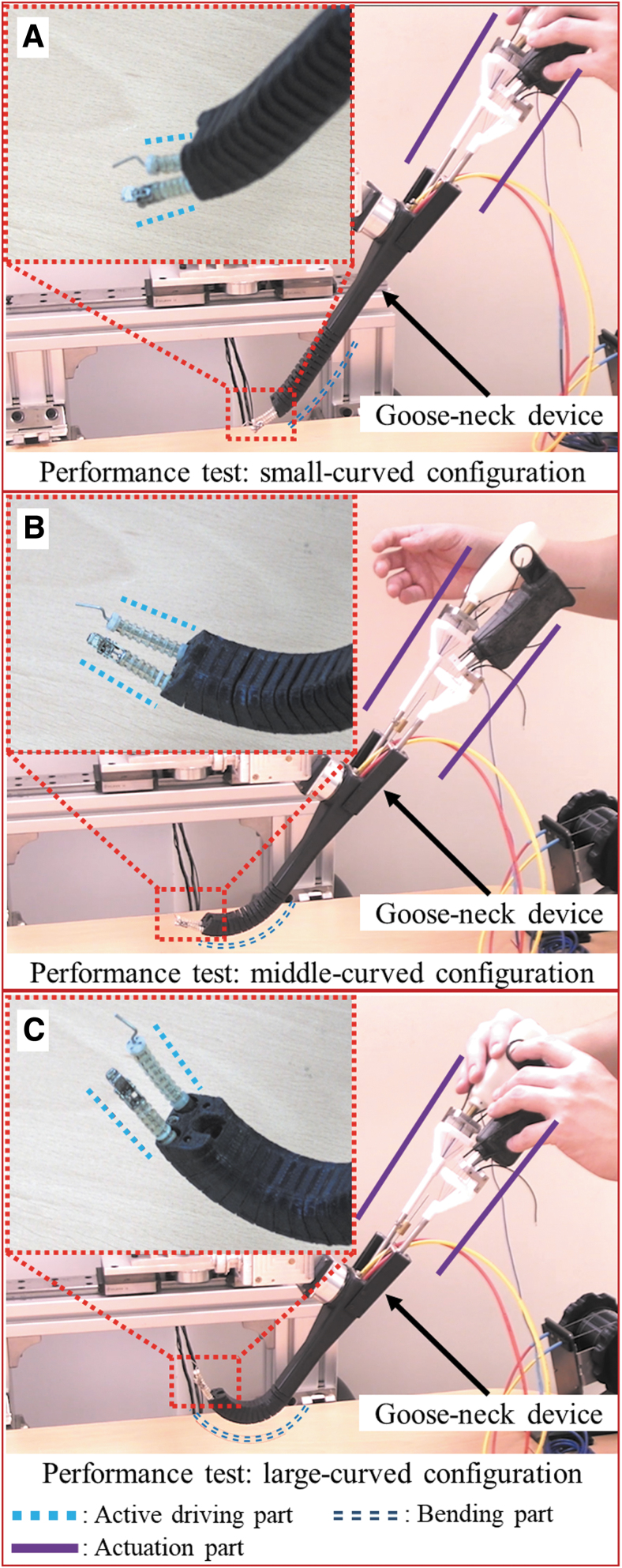
Snapshots for operation of two surgical devices. Operation in different curvature: **(A)** small-curved, **(B)** middle-curved, and **(C)** large-curved configuration. It is observed in **(B, C)** that the active driving part is straight even though the bending part is curved by the gooseneck device. Therefore, the introduction of the helix pattern in the bending part presents outstanding performance in position-level controllability of the active driving part. Color images are available online.

**FIG. 9.**
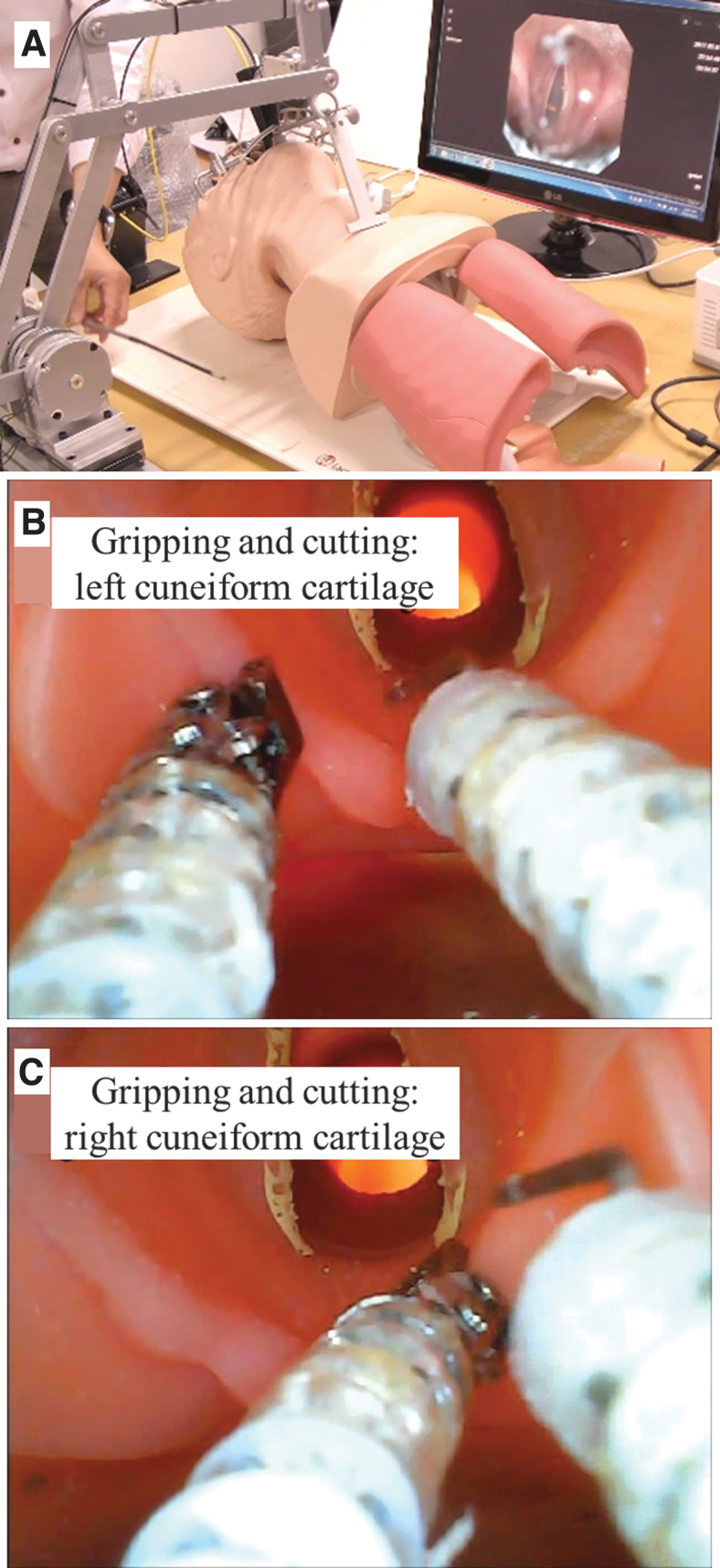
Snapshots for operation of two surgical devices. **(A)** Environment for phantom experiment. The demonstration of gripping and cutting motion for **(B)** left cuneiform cartilage, **(C)** right cuneiform cartilage is conducted. Color images are available online.

Figure 8A–C demonstrates the behavior of the flexible surgical devices under different curvatures of the gooseneck. (Supplementary Movie S2 demonstrates the motion under three kinds of curved environment). The gooseneck part is mounted at the distal end of a passive balancing arm (Fig. 9A). It is observed that the initial configuration of each active driving part maintains straight line although the bending part is curved by the gooseneck device. This implies that there is negligible wire slack due to helix arrangement in wire. Finally, the effectiveness of these surgical devices was tested in a phantom environment in which the tools were used to treat lesions inside the vocal cord area (Fig. 9A–C and Supplementary Movie S2—phantom experiment).

### Experimental procedure and results to verify the effectiveness of the surgical device

Effectiveness of a DNA-helix inspired flexible mechanism will be shown through the experiments of the surgical devices. The performance of surgical device is analyzed in terms of position-level controllability between the input (handle part) and the output (active driving part). With respect to the performance of the position-level controllability, two kinds of experimental results are presented. First, the configuration matching of the handle part between the experiment and the simulation for given position and orientation of the active driving part is presented. Through this experiment, the effectiveness of the simulation for surgical device is verified. Second, the difference of configuration in active driving part is compared between 1 turn and 0 turn helix in the wire guidance part for given orientation of the input (handle part). Through this experiment, the effectiveness of the DNA helix pattern in wire guidance part is verified.

**FIG. 10.**
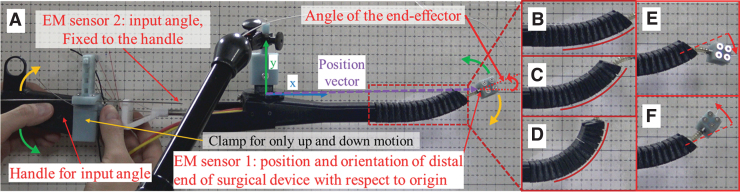
Experimental environment of surgical device. **(A)** General experimental environment (initial configuration). Three kinds of the gooseneck curvature: **(B)** small-curved configuration, **(C)** middle-curved configuration, and **(D)** large-curved configuration. Two kinds of the steering part configuration: **(E)** down configuration and **(F)** up configuration. Color images are available online.

As the common setup for experiments, the experiment was conducted in two-dimensional environment (Fig. 10A). One EM sensor is installed at the handle to measure the input angle, and the other EM sensor is installed at the end tool to measure the x, y positions and the yaw angle of the end effector. The gradation behind the surgical device in snapshot is used to match the configuration between experiment and simulation results. Since the configuration of the active driving part (steering part) is affected by both operation of the handle part and the curvature of the gooseneck mechanism, we consider three different gooseneck curvatures (i.e., small, middle, and large-curved configuration as Fig. 10B–D, respectively) and two kinds of configuration in the active driving part (i.e., up and down configuration as Fig. 10E, F, respectively) in the experiment.

**FIG. 11.**
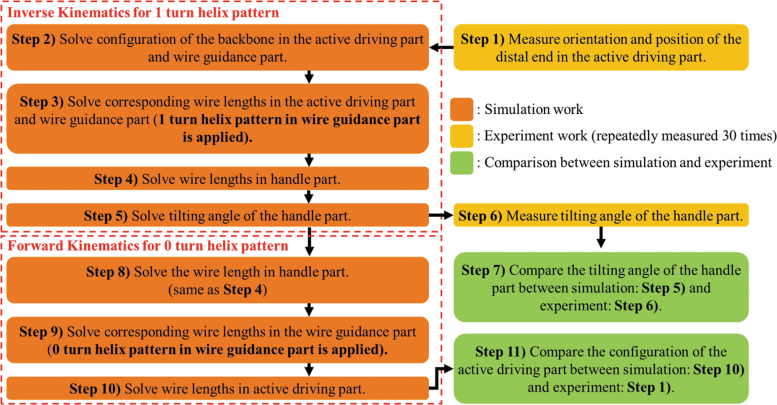
Block diagram of the experimental procedure for configuration matching between simulation and experiment in the surgical device. For the given coordinate of the active driving part, the tilting angle of handle part is solved considering the 1 turn helix in wire guidance part (inverse kinematics). To observe the effectiveness of helix pattern in wire guidance part, the forward kinematics for the 0 turn and 1 turn helix in wire guidance part is solved for given tilting angle in handle part. Finally, their simulated configurations for the active driving part are compared to experimental result to show the effectiveness in position controllability for 1 turn helix case. Color images are available online.

Using the inverse kinematic model (refer to “Inverse Kinematic Modeling of the Surgical Device” section in Supplementary Data) of the surgical device, the effectiveness of the helix turn in the wire guidance part of the surgical device will be shown through the procedure of Figure 11.

Initially, configuration of the gooseneck is set as one of Figure 10B–D. Then, the position and orientation of the end tool in the active driving part are measured using EM sensor 1 (Step 1 in Fig. 11). These values are used to solve the backbone configuration for the active driving part and the bending section in the wire guidance part (Step 2 in Fig. 11). The radius of curvature and the curved angle for both the wire guidance part and the active driving part are solved in this step. Using these values, the length of the straight section of the wire guidance part is calculated, followed by the lengths of two wires in the wire guidance part and the active driving part using geometric design parameters and Equation (5) (Step 3 in Fig. 11).

Since summation of the length of wires in three sections is always constant, the length of wire in the handle part can be obtained and then the tilting angle of the handle part (Steps 4 and 5 in Fig. 11) can be also calculated from the geometry of the handle part. Finally, the simulated tilting angle in the handle part is compared with the experiment value, which is measured by EM sensor 2 (Steps 6 and 7 in Fig. 11). This is the whole procedure of inverse kinematics to verify the effectiveness of the simulation for surgical device.

Next, using the forward kinematic model (refer to “Forward Kinematic Modeling of the Surgical Device” section in Supplementary Data) of the surgical device, the effectiveness of helix wire arrangement in the wire guidance part is shown by comparing the output configurations of active driving part for 1 turn and 0 turn cases in the wire guidance part. Initially, for the same tilting angle in handle part (solved in Step 5 of Fig. 11), the length of wire in the handle part is solved (Step 8 in Fig. 11). And then using the given backbone configuration in the wire guidance part, the lengths of two wires in the wire guidance part can be solved (Step 9 in Fig. 11).

Since the total length of wires in three parts are always constant, the length of wire in the active driving part can be also calculated (Step 10 in Fig. 11). Finally, the simulated configuration of the active driving part is compared with the experimental results (Step 11 in Fig. 11).

The detailed descriptions of the surgical device are shown in Figures S2 to S4 of Supplementary Materials. The proposed experimental procedure, including the inverse and forward kinematics, is iterated for different settings of active driving part and gooseneck configuration. The experiment work (Steps 1 and 6 in Fig. 11) is repeated 30 times.

**FIG. 12.**
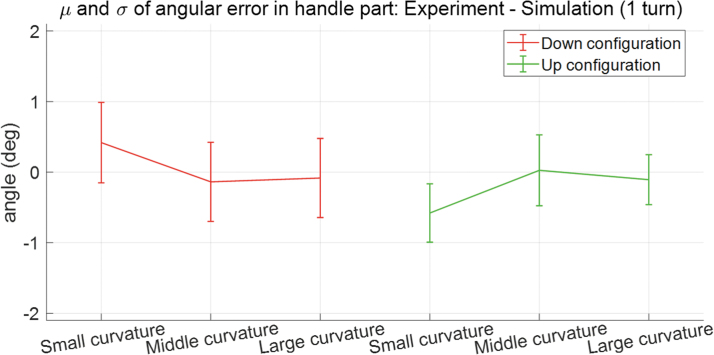
Mean and standard deviation of the angular errors in handle part between experiment and simulation for three kinds of curvature in gooseneck and two kinds of configuration in active driving part for given position and orientation information of distal end in active driving part. The abscissa of the figure denotes the small-curved (small curvature), middle-curved (middle curvature), and large-curved configuration (large curvature) for down and up configuration, respectively. The ordinate of the figure denotes the angular error between the experiment and simulation in handle part. Color images are available online.

To verify the effectiveness of simulation in the inverse kinematic part, the tilting angles in handle part achieved from the experiment and simulation are compared and it is defined as the angular error (Step 7 in Fig. 11). The means and standard deviations of angular error for six cases are presented as Figure 12, and their specific values are presented as Supplementary Table S5. In Figure 12, the middle point of each “I” beam is expressed as the mean of error, and its range is expressed from μ−σ to μ+σ. It is observed from Figure 12 and Supplementary Table S5 that the range of 1σ is less than from −1.0 (deg) to 1.0 (deg) for all cases. These errors are caused by the gap between the diameter of the hole of the gooseneck (7.0 (mm)) and the cross-sectional diameter of bending part (5.6 (mm), see details for surgical device in Supplementary Table S4 for details). However, the errors are acceptable in the operation of the surgical device.

**FIG. 13.**
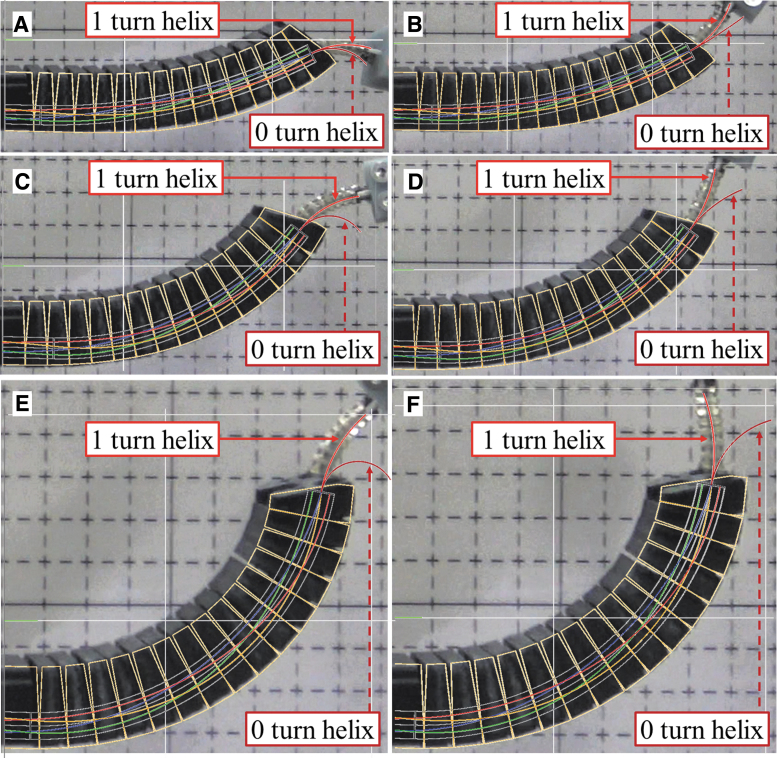
Experimental results of configuration matching between the simulation and experiment for surgical device. The experimental results are presented as the configuration matching between the simulation and experiment for **(A)** down and **(B)** up configuration of small-curved case, **(C)** down and **(D)** up configuration of middle-curved case, and **(E)** down and **(F)** up configuration of large-curved case. In figure, the *red curved lines* denoted as 1 turn helix and 0 turn helix are backbone configurations obtained by the simulation. Color images are available online.

**FIG. 14.**
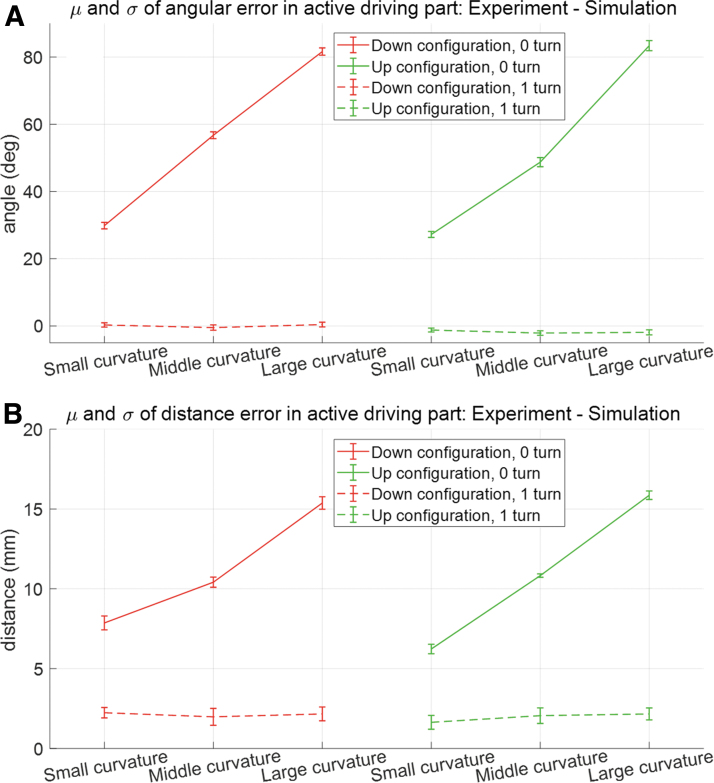
The means and standard deviations for **(A)** angular error of distal end of the active driving part between 0 turn and 1turn in wire guidance part and **(B)** distance error of distal end of the active driving part between 0 turn and 1turn in wire guidance part. It is observed that larger errors for 0 turn case were identified at the active driving part than for 1turn case. The more the gooseneck is curved with large curvature, the more the distance and angular errors occur (maximum mean of error: 15.858 (mm) in distance and 83.374 (deg) in angle). However, for 1 turn case, the angular and distance error is considerably reduced regardless of the curvature in gooseneck mechanism (maximum mean of error: 2.239 (mm) in distance and 2.116 (deg) in angle). Color images are available online.

In the forward kinematic part, to compare configurations in active driving part, the simulation results of the active driving part applying 1 turn and 0 turn helix in the wire guidance part are presented as Figure 13. In Figure 13, the simulation results for configuration of the active driving part and the wire guidance part applying 0 turn helix and 1 turn helix in the wire guidance part are overlapped to the experimental image information to compare the configuration of the active driving part between experiment and simulation. The statistics for distance and angular error at the distal end of the active driving part is expressed as means and standard deviations shown in Figure 14, and their specific values are presented as Supplementary Table S6.

It is observed that large errors were identified at the active driving part for 0 turn case. The more the gooseneck is curved with large curvature, the more the distance and angular errors occur (maximum mean of error: 15.858 (mm) in distance and 83.374 (deg) in angle). In addition, sometimes it could violate the workspace limit of the backbone for active driving part as shown in the simulation result (e.g., Fig. 13C, E). It happens to compensate for the wire slack and strain occurring in wire guidance part for 0 turn case, which is the same phenomenon observed in Figure 5B. Thus, the 0 turn helix design is not recommended. However, for 1 turn case, the angular and distance errors are considerably reduced regardless of the curvature in gooseneck mechanism (maximum mean of error: 2.239 (mm) in distance and 2.116 (deg) in angle). Therefore, the introduction of the helix pattern in the wire guidance part presents outstanding performance in position-level controllability of the end tool in the active driving part. Moreover, the outstanding performance in the position-level controllability of the end tool is demonstrated in simulation of Supplementary Movie S3.

The response time of the surgical device was also analyzed in terms of four delay patterns. It was observed that the response time was fast enough to operate the surgical device in the application. The detailed procedures and results are described in Figures S5 to S7 of Supplementary Materials.

## Conclusion

The main focus of this research is to resolve the wire slack phenomenon, which has been a persistent problem in many engineering fields. DNA helix structure being proposed in this article was applied to resolve such problem.

The main contribution is provision of DNA helix-inspired flexible mechanism that resolves the fundamental wire slack problem. Taking advantage of this helix type wire mechanism, we designed and implemented a surgical device suitable for laryngopharyngeal surgery. The usability of the helix wire has been validated through the controllability in position level. For analysis of the surgical device, the kinematic model of the surgical device is presented. Based on the kinematic model, the configuration considering the relationship between input and output motion was simulated. To verify the effectiveness of helix arrangement of wire in bendable part, two kinds of simulation (with and without helix wire arrangement in bendable part) were compared with the experimental results.

For the purpose of comparison, we fix the configuration of the gooseneck to set the configuration of the wire guidance part. However, in actual operation of the surgical device, the configuration of gooseneck will vary depending upon the anatomical structure of the human body. The design adapting the helix wire will resolve the wire slack problem in a passive manner even if the flexible surgical device bends. The current design is optimized only for Laryngeal surgery, but this concept can be extended to design general endoscope mechanism, especially diverse therapeutic surgical devices requiring highly dexterous motion capability along with considerable payload such as the endoscope, colonoscope, and catheter.

The flexible cylinder manufactured using 3D printing technology is not accurate due to its 3D printer with low resolution (large accumulation height and diameter of the laser beam). However, this can be further improved using 3D printer with high quality. This article mainly focused on kinematic modeling of flexible mechanism, but in future work we seek dynamic modeling of flexible mechanism having general configurations of the backbone with variable curvature caused by an external force. We believe that DNA-helix inspired flexible mechanism driven by multiple wires would open the possibility of many useful applications such as diverse flexible endoscopes and steerable catheter for medical and industrial purposes.

## Supplementary Material

Supplemental data

Supplemental data

Supplemental data

Supplemental data

Supplemental data

Supplemental data

Supplemental data

Supplemental data

Supplemental data

Supplemental data
