# Supplementary material for: DNA-Helix Inspired Wire Routing in Cylindrical Structures and Its Application to Flexible Surgical Devices
Source: Soft Robot. 2022 Apr 19;9(2):337–53. doi: 10.1089/soro.2020.0145 (PMC9057904; doi:10.1089/soro.2020.0145)
Supplement: Supplemental data [file Supp_DataS1.docx]

Supplementary Materials for

**DNA-Helix Inspired Wire Routing in Cylindrical Structures and its Application to Flexible Surgical Devices**

***Construction of the flexible cylinder in fundamental experiment***

The specific design of the flexible cylinder is presented as Figure S1. By inserting a spring between the hole for wire paths and the wire, the friction force can be reduced because the line contact friction is changed to point contact friction as shown in **Figure S1A**. This design is applied to the flexible cylinder for fundamental experiments (Figure 3B-E). In Figure S1B, the specification of the flexible cylinder is described. The details for specifications of the flexible cylinder is presented in ‘Flexible cylinder’ part of Table S4.


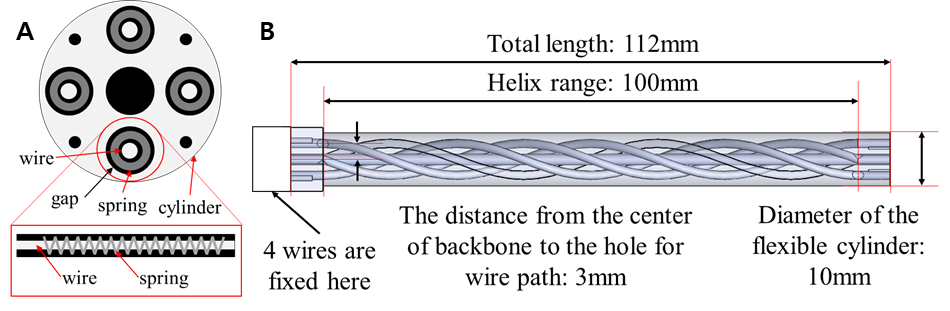


**Fig. S1.** Specific design of the flexible cylinder. (**A**) Front view of the flexible cylinder. Insertion of the spring to the wire path is proposed. (**B**) Side view of the flexible cylinder to present the main properties of length. 2turn helix is chosen as an example.

***Wire lengths of three sections in the surgical device***

To propose the kinematic model of the surgical device, the wire lengths embedded in the surgical device should be analyzed. The kinematic models of 1 turn and 0 turn helix wire pattern used in the wire guidance part are simulated to compare with experimental results. It is noted that the difference in wire length occurs in three sections as shown in **Figure S2A**: the handle part ($L_{u1}\text{ or }L_{u3}$in **Figure S2B**), the wire guidance part ($L_{\text{hx}1}\text{ or }L_{\text{hx}3}$ and $L_{\text{no hx}1}\text{ or }L_{\text{no hx}3}$in **Figure S2C and D**), and the active driving part ($L_{str1}\text{ or }L_{str3}$in Figure S6E). In **Figure S2**, in order to convey the concept clearly, we only consider the planar motion and thus two wires are used to describe the motion of the surgical device. However, it can be extended to 3D case using four wires to control two-directional tilting motion of the active driving part.

Considering that the wire is premised on Kirchhoff’s rod theory (i.e., the wire length is inextensible, and the wire length is constant), the relationship in wire lengths of three sections with and without the helix pattern in the wire guidance part is derived as

(Case of using helix pattern in the wire guidance part)

$L_{u1}+L_{\text{hx}1}+L_{str1}=L_{u1}'+L_{\text{hx}1}'+L_{str1}',$ (total length of wire 1 before and after the motion)

$L_{u3}+L_{\text{hx}3}+L_{str3}=L_{u3}'+L_{\text{hx}3}'+L_{str3}',$ (total length of wire 3 before and after the motion)

(Case of not using helix pattern in the wire guidance part)

$L_{u1}+L_{\text{no hx}1}+L_{str1}=L_{u1}'+L_{\text{no hx}1}'+L_{str1}',$ ( total length of wire 1 before and after the motion)

$L_{u3}+L_{\text{no hx}3}+L_{str3}=L_{u3}'+L_{\text{no hx}3}'+L_{str3}',$ ( total length of wire 3 before and after the motion)

where the motion of the surgical device is defined either when the handle part is rotated or the gooseneck is bent. The wire length before the motion is denoted as $L$ and that after the motion is denoted as $L'$ in general. The numbers ‘1’ and ‘3’ in subscript denote the wire 1 and 3 (red and green line for each section in **Figure S2**, respectively). $L_{u1}\text{ and }L_{u3}$ denote the lengths of wire 1 and 3 between the fixed wall and the rotating link in the handle part before the motion of the handle part, respectively. $L_{u1}'\text{ and }L_{u3}'$ denote the lengths of wire 1 and 3 between the fixed wall and the rotating link in the handle part after the motion, respectively (**Figure S2B**). $(L_{\text{hx1}},L_{\text{hx3}}\text{) and (}L_{\text{no hx1}},\text{ }L_{\text{no hx3}})$ denote the lengths of wire 1 and 3 in the wire guidance part, with and without helix pattern before the motion (straight configuration) (**Figure S2C**), respectively. $(L_{\text{hx1}}',L_{\text{hx3}}')\text{ and (}L_{\text{no hx1}}',L_{\text{no hx3}}')$ denote the lengths of wire 1 and 3 in the wire guidance part, with and without helix pattern after the motion (curved configuration) (**Figure S2D**), respectively.$(L_{str1},L_{str3})$and $(L_{str1}',L_{str3}')$ denote the wire length in the active driving part for wire 1 and 3 before and after the motion, respectively (**Figure S2E**). **Equations S1 to S4** are, respectively, rewritten with respect to the difference in the handle part, the wire guidance part, and the active driving part as

It is assumed that the active driving part always has a constant radius of curvature. For the straight configuration (left figure of **Figure S2B**, **Figure S2C**, left figure of **Figure S2E**), the lengths of wire 1 and 3 are identical in all sections:$L_{u1}=L_{u3}$, $L_{\text{hx1}}=L_{\text{hx3}}$,$L_{\text{no hx1}}=L_{\text{no hx3}}$, and $L_{str1}=L_{str3}$, values of which are given for simulation.

**Fig. S2.** The analysis of three sections where the change in wire lengths occurs: handle part, wire guidance part, and active driving part. The wire guidance part is inserted to the gooseneck. (**A**) The entire configuration of the passive type surgical device in general. (**B**) The length changes of wire 1 and 3 between the fixed wall and the rotating link before and after the motion in handle. (**C**) Wire lengths of wire 1 and 3 in the wire guidance part for with and without helix pattern (before the motion, the gooseneck has no curvature). (**D**) Wire lengths of wire 1 and 3 in the wire guidance part for with and without helix pattern (after the motion, the gooseneck has some curvature). (**E**) The length changes of wire 1 and 3 in the active driving part (steering part) before and after the motion. This is due to motion in the handle part. It is noted that the active driving part does not employ any helix pattern in wires to directly transmit pulling force for creating tilting motion of the active driving part.

***Forward kinematic modeling of the surgical device***

The objective of this section is to solve the position and orientation of the distal end of the active driving part for given input parameters: tilting angle in handle part, curved angle and arc length in the wire guidance part. If these input values are given, the wire lengths in the handle part and in the wire guidance part can be solved. In the wire guidance part, the 0 turn and 1 turn helix pattern of wire arrangement are considered. By using either **Equations S1 and S2** for 1 turn helix case or using the **Equations S3 and S4** for 0 turn helix case, the wire lengths in the active driving part can be solved. Based on this information, the configuration of the active driving part is finally obtained.

As the first step, the wire lengths in the handle part after the motion are analyzed. The geometric structure of the handle part is described in **Figure S3**, where $\alpha$denotes the tilting angle of the handle part. $W_{u}\text{, }L_{w2u},r_{u},\text{ and }r_{w}$denote the half width of the universal joint, the distance between the wall and center of rotation joint, the radius between the center of rotation joint and the wire hole in universal joint, and the distance between the center of rotation joint and the wire hole in the wall, respectively. $\underline{p}_{u11},\underline{p}_{u12},\underline{p}_{u31},\text{ and }\underline{p}_{u32}$ denote the position vectors of the wire holes at the universal joint and the wall, respectively. For the given tilting angle $\alpha$, the wire lengths ($L_{u1}'\text{ and }L_{u3}'$) in the handle part after the motion are solved as follows

$$L_{u1}'\text{ }=\sqrt{(\underline{p}_{u11}-\underline{p}_{u12})^{T}(\underline{p}_{u11}-\underline{p}_{u12})}=\sqrt{(-L_{w2u}+W_{u}\cos\alpha+r_{u}\sin\alpha)^{2}+(r_{w}+W_{u}\sin\alpha-r_{u}\cos\alpha)^{2}}$$

.

**Fig. S3.** Geometric description about the length of the wire 1 and 3 to create the tilting angle of the universal joint in the handle part of the surgical device. Geometric relationship between the tilting angle of the universal joint and the lengths of wire 1 and 3 are presented. It is noted that the wire lengths from the wall to the straight section of the wire guidance part are constant regardless of tilting motion in the handle part. And thus the length change in the handle part directly affects the length changes in the curved wire guidance part and the active driving part.

In the next step, the configuration of the backbone of the surgical device, which covers the wire guidance part and the active driving part, is analyzed as shown in **Figure S4**, where$R_{gui2}(=L_{b,gui2}/\theta_{gui})$ and $R_{str}(=L_{b,str}/\theta_{str})$denote the radius curvature in the wire guidance part and the active driving part, respectively. $L_{b,gui2},L_{b,str},\theta_{gui},\text{ and }\theta_{str}$ denote the backbone length in the wire guidance part and the active driving part, and the curved angle in the wire guidance part and the active driving part, respectively. In the wire guidance part, there exists a straight part and a curved part as shown in **Figure S2D**. It is assumed that the radius of curvature in the curved part of the gooseneck is constant. The transformation matrix$g_{e}$at the distal end of the active driving part will be expressed relative to the coordinate system $g_{b,gui2}$located at the starting point of the curved section in the wire guidance part. It can be derived by applying the content of ‘Generalized kinematic modeling’ section in the main manuscript. In order to facilitate the inverse kinematics, we set the starting point of the curved section in the wire guidance part as the center of the reference coordinate system. Then, $g_{b,gui2}$ can be set as $I_{4\times4}$. The position vector to the center of curvature, its angular velocity vector, and twist in the curved part of the wire guidance part are provided as

$\underline{q}_{b,gui2}=\left[\begin{matrix} 0 & R_{gui2} & 0 \end{matrix}\right]^{T},$ $\underline{w}_{b,gui2}=\left[\begin{matrix} 0 & 0 & 1 \end{matrix}\right]^{T}\text{,}$and $\xi_{b,gui2}=\left[\begin{matrix} R_{gui2} & 0 & 0 & 0 & 0 & 1 \end{matrix}\right]^{T}.$

Those in the active steering part are

$\underline{q}_{b,str}=\left[\begin{matrix} 0 & -R_{str} & 0 \end{matrix}\right]^{T},$ $\underline{w}_{b,str}=\left[\begin{matrix} 0 & 0 & -1 \end{matrix}\right]^{T}\text{,}$and $\xi_{b,str}=\left[\begin{matrix} -R_{str} & 0 & 0 & 0 & 0 & -1 \end{matrix}\right]^{T}.$

Therefore, the transformation matrix $g_{b,str}$at the distal end of the wire guidance part relative to $g_{b,gui2}$ and the transformation matrix $g_{e}$ at the distal end of the active driving part relative to $g_{b,str}$ are solved as

where

$$Rot_{gui2}=\left[\begin{matrix} \cos(\theta_{gui}) & -\sin(\theta_{gui}) & 0 \\ \sin(\theta_{gui}) & \cos(\theta_{gui}) & 0 \\ 0 & 0 & 1 \end{matrix}\right],$$

$$\underline{p}_{gui2}=\left[\begin{matrix} R_{gui2}\sin(\theta_{gui}) \\ R_{gui2}(1-\cos(\theta_{gui})) \\ 0 \end{matrix}\right],$$

$$Rot_{str}=\left[\begin{matrix} \cos(\theta_{str}) & \sin(\theta_{str}) & 0 \\ -\sin(\theta_{str}) & \cos(\theta_{str}) & 0 \\ 0 & 0 & 1 \end{matrix}\right],$$

and

$$\underline{p}_{str}=\left[\begin{matrix} R_{str}\sin(\theta_{str}) \\ -R_{str}(1-\cos(\theta_{str})) \\ 0 \end{matrix}\right].$$

Finally, the transformation of $g_{e}$relative to $g_{b,gui2}$ is solved as

$$g_{e}(\theta_{gui2},\theta_{str})=e^{\hat{\xi}_{b,gui2}\text{ }\theta_{gui}}e^{\hat{\xi}_{b,str}\text{ }\theta_{str}}=\left[\begin{matrix} Rot_{e} & \underline{p}_{e} \\ 0 & 1 \end{matrix}\right],$$

where

$$Rot_{e}=\left[\begin{matrix} \cos(\theta_{gui}-\theta_{str}) & -\sin(\theta_{gui}-\theta_{str}) & 0 \\ \sin(\theta_{gui}-\theta_{str}) & \cos(\theta_{gui}-\theta_{str}) & 0 \\ 0 & 0 & 1 \end{matrix}\right]=Rot(\underline{\hat{z}},\Phi_{E}),$$

$$\underline{p}_{e}=\left[\begin{matrix} R_{gui2}\sin(\theta_{gui})+R_{str}\sin(\theta_{gui})-R_{str}\sin(\theta_{gui}-\theta_{str}) \\ R_{gui2}-R_{gui2}\cos(\theta_{gui})-R_{str}\cos(\theta_{gui})+R_{str}\cos(\theta_{gui}-\theta_{str}) \\ 0 \end{matrix}\right]=\left[\begin{matrix} x_{e} \\ y_{e} \\ 0 \end{matrix}\right],$$

where $\Phi_{E}(=\theta_{gui}-\theta_{str})$denotes the output angle of the surgical tool.

In the third step, the wire lengths in the wire guidance part can be obtained using the information of the curved angle and arc length both in the wire guidance part ($\theta_{gui}\text{ and }L_{b,gui2}$). For both helix and no helix cases, the curved configuration of the wire guidance part is given by fixing the gooseneck configuration. That implies that $\theta_{gui}\text{ and }L_{b,gui2}$is given. For the helix case, the wire length of the curved wire guidance part can be solved by using **Equation 5**. For no helix cases, the left figure of **Figure S4** describing the geometry of the double curvature model given by

$$\theta_{gui}=\frac{L_{b,gui2}}{R_{gui2}}=\frac{L_{\text{no hx32}}'}{R_{gui2}-r}=\frac{L_{\text{no hx12}}'}{R_{gui2}+r}$$

is used to calculate the length of wire. In **Figure S4**, $r$ denotes the distance between the wire and the backbone in the wire guidance part, $L_{\text{no hx12}}'$ $\text{and }L_{\text{no hx32}}'$ denote the arc length of the wire 1 and 3 in the curved section (after the motion), and $L_{\text{no hx11}}'\text{ }(=L_{\text{no hx11}}\text{) and }L_{\text{no hx31}}'\text{ }(=L_{\text{no hx31}}\text{)}$denote the arc length of the wire 1 and 3 in the straight section, respectively. Since we can solve the $L_{\text{no hx12}}'$ $\text{and }L_{\text{no hx32}}'$ through **Equation S14**, the total length of wire 1 and 3 in the wire guidance part without helix ($L_{\text{no hx1}}'$,$L_{\text{no hx3}}'$) can be solved as

and its difference is

$$L_{\text{no hx1}}'-L_{\text{no hx3}}'=\frac{2rL_{b,gui2}}{R_{gui2}}.$$

In the fourth step, the wire lengths in the active driving part can be obtained since the wire lengths in other sections are already obtained. **Equations S3 and S4** for no helix case and **Equations S1 and S2** for helix case will be used for this. Using these information, the curved geometry (i.e., the curved angle and arc length: $\theta_{str}\text{ and }L_{b,str}$) of the active driving part can be obtained as described in **Figure S4**. For no helix case, the curvature model of the active driving part can be obtained using the relationship given by

$$\theta_{str}=\frac{L_{str3}'}{R_{str}+r}=\frac{L_{b,str}}{R_{str}}=\frac{L_{str1}'}{R_{str}-r}.$$

from which the curvature of radius in the active driving part$R_{str}$is solved as

$$R_{str}=r\cdot\frac{L_{str3}'+L_{str1}'}{L_{str3}'-L_{str1}'}.$$

since the backbone length in the active driving part $L_{b,str}$is known. Next, the curved angle of the active driving part $\theta_{str}$ can be obtained as $\theta_{str}=L_{b,str}/R_{str}.$ For 1 turn helix case, the curvature model of the active driving part can be obtained with the same manner. It is noted that this section corresponds to Step 8, 9, and 10 for 0 turn helix in the wire guidance part in **Figure 11**.

**Fig. S4.** Geometric description about the length of the wire 1 and 3 in the wire guidance part and the active driving part. A double curvature model of the wire guidance part and the active driving part is presented. The above figure only describes the case of no helix pattern in the wire guidance part, although we can also consider the case of helix pattern. When the surgical device is inserted to the hole of the goose-neck, there exist a straight part and a curved part in the surgical device. If the arc lengths of straight and curved parts and its radius of curvature in the wire guidance part are known, the lengths of wire 1 and 3 in the wire guidance part can be solved. It is noted that the active driving part is attached to the distal end of the wirer guidance part. The active driving part deflects by pulling wires. However, the direction of curvature is opposite to that of the wire guidance part since the whole system is trying to maintain the length of wire.

***Inverse kinematic modeling of the surgical device***

The objective of this section is to solve the entire configuration of the surgical tool in **Figure S4** for the given position and orientation of the distal end for the active driving part. As described in forward kinematic section, the transformation $g_{e}$ of the active driving part is expressed relative to the coordinate system $g_{b,gui2}$ at starting point of curvature in the wire guidance part. If $\theta_{gui}\text{, }L_{b,gui2}$ $,\text{ and }\theta_{str}$ are solved, wire lengths in the active driving part ($L_{str1}\text{' and }L_{str3}'$) and the wire guidance part can be solved. In the wire guidance part, the length of wire including 0 turn and 1 turn helix pattern of wire arrangement ($L_{\text{no hx1}}',L_{\text{no hx3}}',$ $L_{\text{hx1}}',\text{ and }L_{\text{hx3}}'$) are solved. By using **Equations S1 and S2** for 1 turn helix case or using the **Equations S3 and S4** for 0 turn helix case, the resultant wire lengths in the handle part are solved and then the tilting angle $\alpha$ in the handle part is finally solved. in terms of three variables$\theta_{gui}\text{, }L_{b,gui2},\text{ and }\theta_{str}$

As the first step, $\theta_{gui}\text{, }L_{b,gui2},\text{ and }\theta_{str}$ will be solved for given the values of $g_{e}$with respect to $g_{b,gui2}$. By inserting the geometric information $R_{gui2}=L_{b,gui2}/\theta_{gui}$and $R_{str}=L_{b,str}/\theta_{str}$into $\underline{p}_{e}$and $Rot_{e}$of **Equation S13**, the coordinates at the distal end of the active driving part is rewritten as

$$\begin{matrix} x_{e}=\frac{L_{b,gui2}}{\theta_{gui}}\sin(\theta_{gui})+\frac{L_{b,str}}{\theta_{str}}\sin(\theta_{gui})-\frac{L_{b,str}}{\theta_{str}}\sin(\theta_{gui}-\theta_{str}), \\ y_{e}=\frac{L_{b,gui2}}{\theta_{gui}}-\frac{L_{b,gui2}}{\theta_{gui}}\cos(\theta_{gui})-\frac{L_{b,str}}{\theta_{str}}\cos(\theta_{gui})+\frac{L_{b,str}}{\theta_{str}}\cos(\theta_{gui}-\theta_{str}), \\ \Phi_{E}=\theta_{gui}-\theta_{str}. \end{matrix}$$

It is noted that the length of the active driving part $L_{b,str}$ is given as a constant value. Since there exist three equations and three unknown variables ($\theta_{gui}\text{, }L_{b,gui2},\text{ and }\theta_{str}$), these variables can be solved. Because of nonlinear characteristic of the first and second equations in **Equation S20**, $\theta_{gui}\text{, }L_{b,gui2},\text{ and }\theta_{str}$ are solved by the numerical solver tool. Once$\theta_{gui}\text{, }L_{b,gui2},\text{ and }\theta_{str}$are solved, $R_{gui2},\text{ and }R_{str}$can be calculated.

In the second step, the wire lengths $L_{\text{hx12}}', L_{\text{hx32}}', L_{\text{no hx12}}', L_{\text{no hx32}}', L_{str1}',\text{ and} \text{ }L_{str3}\text{'}$ (in the curved section of the wire guidance part considering 1 turn and 0 turn helix case, and the active driving part) can be solved. $L_{str1}\text{' and }L_{str3}'$are solved by using **Equation S18**. For 1 turn helix case, the wire lengths ($L_{\text{hx1}}'\text{ and }L_{\text{hx3}}'$) in the wire guidance part can be solved by using Equation 1. For 0 turn helix case, the length of wires ($L_{\text{no hx1}}'\text{ and }L_{\text{no hx3}}'$) can be obtained from **Equations S15 and S16** since the lengths of wires in the straight part of the wire guidance part ($L_{\text{no hx11}}'\text{ and }L_{\text{no hx31}}'$) are constant.

In the third step, the length of wires in the handle part ($L_{u1}\text{' and }L_{u3}'$) are solved by using **Equations S1 and S2** for helix pattern in the wire guidance part and **Equations S3 and S4** for no helix pattern in the wire guidance part. For the solved $L_{u1}\text{' and }L_{u3}'$, the tilting angle $\alpha$ in **Figure S3** can be solved by using **Equations S9 and S10** as follows.

$$\begin{matrix} (L_{u3}')^{2}-\text{(}L_{u1}')^{2}\text{ }=4\sin\alpha(r_{u}L_{w2u}-r_{w}W_{u}) \\ \therefore\alpha=\sin^{-1}\left(\frac{(L_{u3}')^{2}-\text{(}L_{u1}')^{2}}{4(r_{u}L_{w2u}-r_{w}W_{u})}\right) \end{matrix}$$

where $\alpha\in(-\pi/2,\text{ }\pi/2)$, which is constrained by design. It is noted that this section corresponds to Step 2, 3, 4, and 5 for 1 turn helix in the wire guidance part in **Figure 11**.

***Response time of the surgical device between input and output***

As the second category for the performance of surgical device, the measurement of response time between the input (handle part) and output (active driving part) is dealt. The effect of wire slack on the response time is described as. In **Figure S5**, the response time is calculated as $t_{2}-t_{1}$. The more slacks wire in the hole, the more response time. Since the wire slack and response time are closely related, the measurement of the response time is introduced.

As a measurement method of the response time, the surgical device is initially set on its initial configuration as shown in **Figure S6**, then the handle is repeatedly moved up (about +13 (deg)) and down (about -13 (deg)), respectively. The input is given as the handle motion and the output is given as the motion of the active driving part. One cycle consists of down to up and up to down motion. For one cycle of these repeated moves, 4 delay patterns (namely, delay pattern 1, 2, 3, and 4) can be analyzed: initial rise delay from down configuration, settling delay to up configuration, initial fall delay from up configuration, and settling delay to down configuration as shown in **Figure S6**. Each delay patterns are measured as time differences of $t_{out,10}-t_{in,10}$ at 10% rising time (delay pattern 1) and $t_{out,90}-t_{in,90}$ at 90% rising time (delay pattern 2), $t_{out,10}-t_{in,10}$at 10% fall time (delay pattern 3) and $t_{out,90}-t_{in,90}$ at 90% fall time (delay pattern 4), respectively (**Figure S6**). The delay pattern 1 and 3 are caused by the phenomenon presented in **Figure S5**.

The experiment for 4 delay patterns are repeated 30 times and the statistical results of 4 delay patterns for each curvature are presented (**Figure S7A-C**). It is observed that the initial rise and fall response time (delay pattern 1 and 3) are larger than the settling response time (delay pattern 2 and 4). The response time increases further for more curved cases, but its medians do not exceed 0.1(sec) in all cases. It was verified that the response time between the handle operation as input and the movement in the active driving part as output is fast enough for surgical operation.


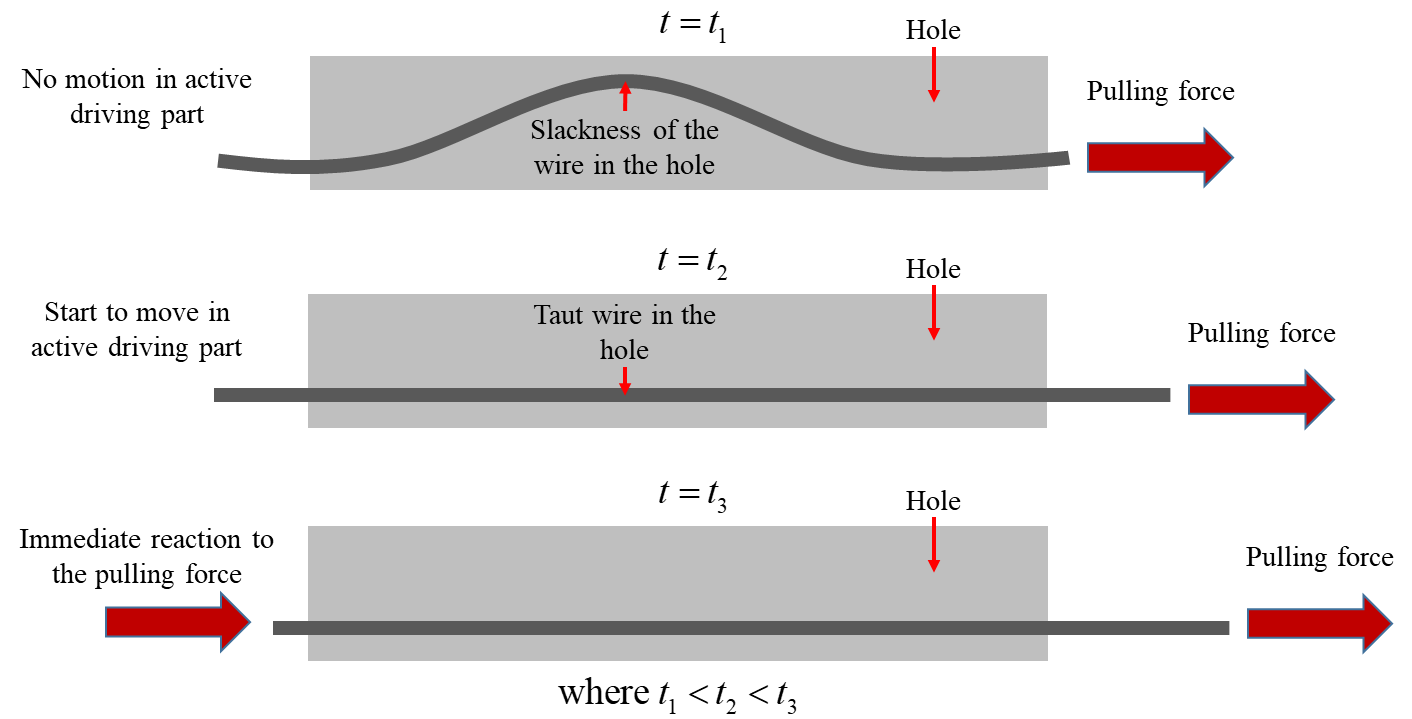


**Fig. S5.** The process for transmission of the pulling force to the active driving part.


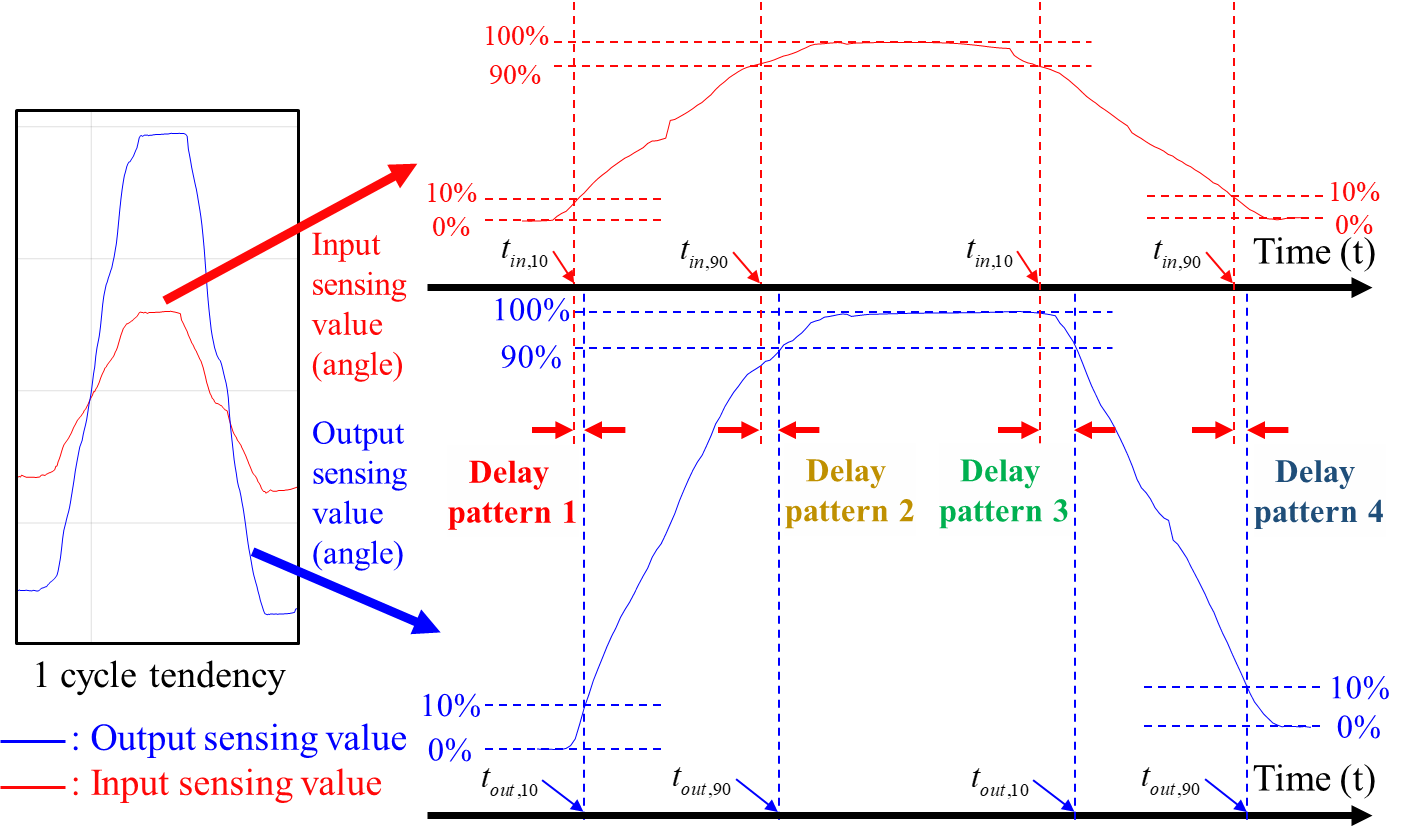


**Fig. S6.** Measurement of four kinds of delay patterns.


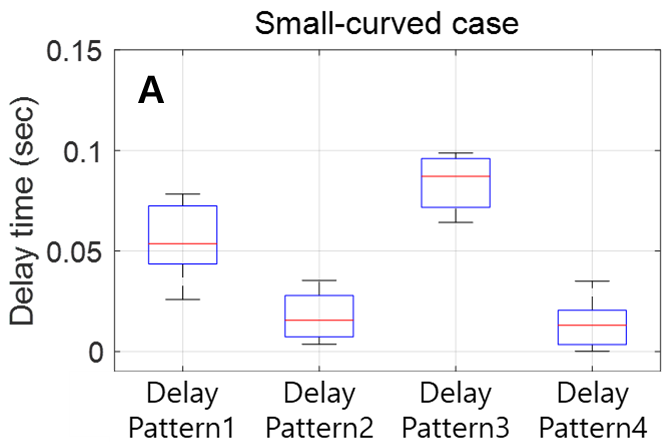

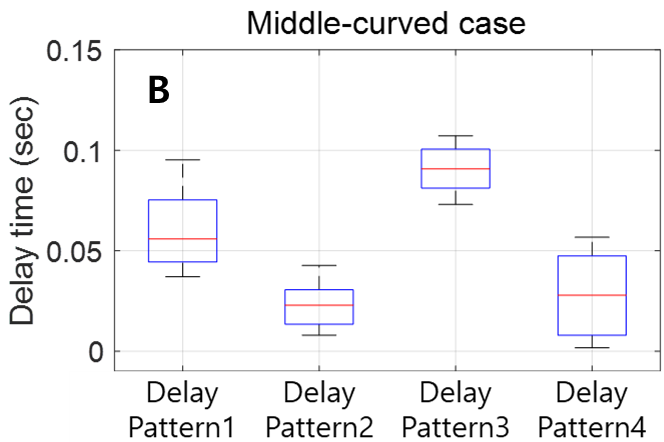


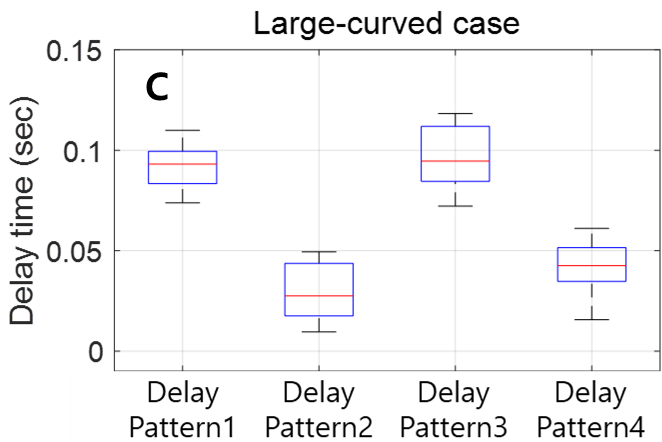


**Fig. S7.** The boxplots of response time for each delay pattern between input and output of the surgical device (**A**) small-curved case, (**B**) middle-curved case, and (**C**) large-curved case.

***The 3D printer and its printing materials***

The 3D printing technology was applied to the construction of the experimental environment for fundamental experiment, surgical device. The details of 3D printer, materials, and applications to experimental environment are presented in **Table S4**.

***The electromagnetic sensor***

The electromagnetic(EM) sensor was used to measure the position and orientation of rigid bodies throughout all experiments (**Figure 3M** in the fundamental experiment, **Figure 10** in the experiment of surgical device). The EM sensor model is Aurora of Northern Digital Inc., and the EM sensor system consists of the system control unit, field generator, sensor interface unit, and PC. With respect to the sensor interface unit, the EM sensor measures the 6-DOF, and the EM sensor’s accuracy in position and angle are 0.48(mm) and 0.3(deg), respectively. The resolution in position and angle are 0.001mm and 0.001(deg), respectively. The measurement volume of the field generator is a cube of 500$\times$500$\times$500 (mm). The sampling time for sensing data is 40Hz. With respect to the system control unit, the host interface is RS-232 and the maximum data rate is 115kbaud. CPU specification of the PC is 3.5GHz and 8GB RAM.

***The PC for simulation***

The PC is used to simulate the length of helix wire to match configuration between simulation and experimental results (**Figure 2 and Figure 4** in fundamental experiment, and **Figure 12 and Figure 14** in surgical device). The CPU specification of the PC is 3.4GHz and 16GB RAM. Matlab R2019b was used for calculation and plot for figures and tables.
