# Supplementary material for: DNA-Helix Inspired Wire Routing in Cylindrical Structures and Its Application to Flexible Surgical Devices
Source: Soft Robot. 2022 Apr 19;9(2):337–53. doi: 10.1089/soro.2020.0145 (PMC9057904; doi:10.1089/soro.2020.0145)
Supplement: Supplemental data [file Supp_TableS1.docx]

Table S1. Specifications of the experimental environment for fundamental experiment.

| Details for fundamental experiment | | | Specification | |
| --- | --- | --- | --- | --- |
| Flexible cylinder | Total length | 112 mm | |  |
|  | Helix range | 100 mm | |  |
|  | 4 kinds of helix pitch | Infinity (0 turn), 100mm (1 turn), 66.67mm (1.5 turn), 50mm (2 turn) | |  |
|  | Diameter of cross section of flexible cylinder | 10 mm | |  |
|  | The distance from the center of backbone to the hole for wire path | 3 mm | |  |
|  | Diameter of hole | 1.60 mm | |  |
|  | Outer/inner diameter of the inserted springs | 1.33mm/ 0.81mm | |  |
| Wire | Cross-sectional diameter of the wire | 0.54mm | |  |
| Rails | Four kinds of curvature radius of rail elements | Infinity, 200mm, 100mm, 50mm | |  |
|  | Common arc length of the rail elements | 50mm | |  |
