# Supplementary material for: DNA-Helix Inspired Wire Routing in Cylindrical Structures and Its Application to Flexible Surgical Devices
Source: Soft Robot. 2022 Apr 19;9(2):337–53. doi: 10.1089/soro.2020.0145 (PMC9057904; doi:10.1089/soro.2020.0145)
Supplement: Supplemental data [file Supp_TableS2.docx]

**Table S2.** Details of 3D printer and materials. The 3D printer, materials, and applications to experimental environment are presented.

| Details of 3D printer | | Specification |
| --- | --- | --- |
| 3D printer | Model | XFAB2000 of DWS |
|  | Printing method | laser stereolithograhpy type |
|  | Diameter of laser beam | 0.07mm |
|  | Diameter and height of working area (cylindrical shape) | 180mm/180mm |

| Details of materials used in 3D printer | | | Specification | Application |
| --- | --- | --- | --- | --- |
| Materials | Flexa 693 | Shore durometer (Type D) | 35 | Design of flexible cylinder in the fundamental experiment  (Figure 3B-E) |
|  |  | Accumulation height | 0.1mm |  |
|  | Invicta 917  (gray colored) | Shore durometer (Type D) | 81~83 | Design of flexible cylinder in the fundamental experiment  (Figure 3, F, I, J, K, L) |
|  |  | Accumulation height | 0.1mm |  |
|  |  | Tensile Strength/  Tensile Modulus | 30~50 MPa/  1350~2400 MPa |  |
|  |  | Flexural Strength/  Flexural modulus | 60~95 MPa/  1500~2200 MPa |  |
|  | Invicta 977  (transparent) | Shore durometer (Type D) | 86~88 | Design of flexible cylinder in the fundamental experiment  (Figure 3, G, H, J, K) |
|  |  | Accumulation height | 0.1mm |  |
|  |  | Tensile Strength/  Tensile Modulus | 35~45 MPa/  1750~2150 MPa |  |
|  |  | Flexural Strength/  Flexural modulus | 80~110 MPa/  1850~2600 MPa |  |
|  | Therma 294  (light blue colored) | Shore durometer (Type D) | 88~91 | Design of holders in the experiment of surgical device (light blue material in Figure S2) |
|  |  | Accumulation height | 0.07mm |  |
|  |  | Tensile Strength/  Tensile Modulus | 20~45 MPa/  1800~2900 MPa |  |
|  |  | Flexural Strength/  Flexural modulus | 55~125 MPa/  2000~3400 MPa |  |
