# Supplementary material for: DNA-Helix Inspired Wire Routing in Cylindrical Structures and Its Application to Flexible Surgical Devices
Source: Soft Robot. 2022 Apr 19;9(2):337–53. doi: 10.1089/soro.2020.0145 (PMC9057904; doi:10.1089/soro.2020.0145)
Supplement: Supplemental data [file Supp_TableS3.docx]

Table S3. Root mean square (RMS) of simulation, experiment, and difference between simulation and experiment for length variation of 4 wire lumens. For 4 kinds of helix turn of wire lumen in flexible cylinder and 6 kinds of different guide rail setting, the RMS of simulation, experiment, and their errors for length variation of 4 wire lumens are presented.

| Unit: mm | 0 turn | | 1 turn | | 1.5 turn | | 2 turn | |
| --- | --- | --- | --- | --- | --- | --- | --- | --- |
| RMS of length variation of 4 wire lumens between two different guide rails | simulation [A]/  experiment [B] | [A] - [B] | simulation [A]/  experiment [B] | [A] - [B] | simulation [A]/  experiment [B] | [A] - [B] | simulation [A]/  experiment [B] | [A] - [B] |
| Fig. 3  (G -F) | 1.061/  1.056 | 4.592$\cdot10^{-3}$ | 1.897$\cdot10^{-4}$/  2.884$\cdot10^{-2}$ | -2.865$\cdot10^{-2}$ | 0.217/  0.232 | -1.570$\cdot10^{-2}$ | 6.550$\cdot10^{-4}$/  2.527$\cdot10^{-2}$ | -2.461$\cdot10^{-2}$ |
| Fig. 3  (H -F) | 2.121/  2.105 | 1.620$\cdot10^{-2}$ | 7.591$\cdot10^{-4}$/  2.776$\cdot10^{-2}$ | -2.700$\cdot10^{-2}$ | 0.433/  0.464 | -3.095$\cdot10^{-2}$ | 2.621$\cdot10^{-3}$/  2.947$\cdot10^{-2}$ | -2.685$\cdot10^{-2}$ |
| Fig. 3  (I -F) | 4.243/  4.213 | 2.934$\cdot10^{-2}$ | 3.042$\cdot10^{-3}$/  2.777$\cdot10^{-2}$ | -2.473$\cdot10^{-2}$ | 0.866/  0.856 | 9.911$\cdot10^{-3}$ | 1.050$\cdot10^{-2}$/  3.559$\cdot10^{-2}$ | -2.509$\cdot10^{-2}$ |
| Fig. 3  (J -F) | 0.530/  0.545 | -1.512$\cdot10^{-2}$ | 0.332/  0.336 | -3.931$\cdot10^{-3}$ | 0.153/  0.161 | -7.629$\cdot10^{-3}$ | 3.275$\cdot10^{-4}$/  2.214$\cdot10^{-2}$ | -2.181$\cdot10^{-2}$ |
| Fig. 3  (K -F) | 1.061/  1.055 | 5.730$\cdot10^{-3}$ | 0.664/  0.676 | -1.230$\cdot10^{-2}$ | 0.306/  0.315 | -8.899$\cdot10^{-3}$ | 1.311$\cdot10^{-3}$/  2.652$\cdot10^{-2}$ | -2.521$\cdot10^{-2}$ |
| Fig. 3  (L -F) | 2.121/  2.105 | 1.683$\cdot10^{-2}$ | 1.327/  1.327 | -1.903$\cdot10^{-4}$ | 0.613/  0.619 | -6.618$\cdot10^{-3}$ | 5.250$\cdot10^{-3}$/  2.831$\cdot10^{-2}$ | -2.306$\cdot10^{-2}$ |
