# Supplementary material for: DNA-Helix Inspired Wire Routing in Cylindrical Structures and Its Application to Flexible Surgical Devices
Source: Soft Robot. 2022 Apr 19;9(2):337–53. doi: 10.1089/soro.2020.0145 (PMC9057904; doi:10.1089/soro.2020.0145)
Supplement: Supplemental data [file Supp_TableS4.docx]

Table S4. Specifications of the experimental environment for the surgical device.

| Details for surgical device | | Specification |
| --- | --- | --- |
| Surgical device | The length of the helix wire guidance part | 150mm  (with 1 turn helix) |
|  | The cross-sectional diameter of the bendable wire guidance part | 5.6mm |
|  | The length of the active driving part | 20mm |
|  | The cross-sectional diameter of the active driving part | 5.6mm |
|  | Minimum curvature radius of the active driving part (large-curved) | 6.5mm |
|  | The length of the straight passive part | 158mm |
|  | The cross-sectional diameter of the straight passive part | 6.0mm |
|  | The cross section diameter of the wire | 0.54mm |
|  | The distance from the backbone to the wire | 1.9mm |
|  | The diameter of wire arrangement in universal joint | 18mm |
| Gooseneck mechanism | The length of the goose-neck section | 95mm |
|  | The diameter of hole for insertion of end-effector | 7.0mm |
