# Supplementary material for: DNA-Helix Inspired Wire Routing in Cylindrical Structures and Its Application to Flexible Surgical Devices
Source: Soft Robot. 2022 Apr 19;9(2):337–53. doi: 10.1089/soro.2020.0145 (PMC9057904; doi:10.1089/soro.2020.0145)
Supplement: Supplemental data [file Supp_TableS5.docx]

**Table S5.** The means and standard deviations of error for 6 cases for tilting angles in handle part between simulation and experiment. It is observed that range of 1$\sigma$ for error ranges are less than from -1.0 (deg) to 1.0 (deg). These errors are caused by the gap between the diameter of the hole of the goose-neck (7.0mm) and the cross-sectional diameter of wire guidance part (5.6mm). However, the errors are acceptable in the operation of the surgical device.

| Unit: deg | Down configuration | | | Up configuration | | |
| --- | --- | --- | --- | --- | --- | --- |
| Angular errors in handle part:  experiment - simulation (1 turn) | Small  curvature | Middle  curvature | Large  curvature | Small  curvature | Middle  curvature | Large  curvature |
| Simulation values | -11.271 | -10.696 | -10.552 | 12.923 | 10.649 | 12.430 |
| $\mu$ of errors | 0.419 | -0.138 | -0.083 | -0.579 | 0.026 | -0.107 |
| $\sigma$ of error | 0.570 | 0.560 | 0.561 | 0.413 | 0.503 | 0.354 |
| Range of 1$\sigma$ for error | -0.151 ~ 0.989 | -0.698 ~ 0.422 | -0.644 ~ 0.478 | -0.992 ~ -0.166 | -0.477 ~ 0.529 | -0.461 ~ 0.247 |
