# Supplementary material for: DNA-Helix Inspired Wire Routing in Cylindrical Structures and Its Application to Flexible Surgical Devices
Source: Soft Robot. 2022 Apr 19;9(2):337–53. doi: 10.1089/soro.2020.0145 (PMC9057904; doi:10.1089/soro.2020.0145)
Supplement: Supplemental data [file Supp_TableS6.docx]

**Table S6** The means and standard deviations of angular and distance error between experiment and simulation for 6 cases. It is observed that large errors were identified at the active driving part for 0 turn case. The more the goose-neck is curved with large curvature, the more the distance and angular errors occur (maximum mean of error: 15.858(mm) in distance and 83.374(deg) in angle). However, for 1 turn case, the angular and distance error is considerably reduced (maximum mean of error: 2.239 (mm) in distance and 2.116 (deg) in angle).

| Unit: deg | Down configuration | | | Up configuration | | |
| --- | --- | --- | --- | --- | --- | --- |
| Angular error in active driving part:  experiment - simulation (0 turn)/  experiment - simulation (1 turn) | Small  curvature | Middle  curvature | Large  curvature | Small  curvature | Middle  curvature | Large  curvature |
| $\mu$ of errors | 29.841/  0.303 | 56.729/  -0.487 | 81.627/  0.422 | 27.224/  -1.214 | 48.738/  -2.1155 | 83.374/  -1.898 |
| $\sigma$ of error | 0.963/  0.636 | 1.026/  0.797 | 1.083/  0.700 | 0.888/  0.574 | 1.357/  0.668 | 1.506/  0.759 |
| Range of 1$\sigma$ for error | 28.878 ~ 39.804/  -0.333 ~ 0.938 | 55.703 ~ 57.755/  -1.283 ~ 0.310 | 80.544 ~ 82.710/  -0.279 ~ 1.122 | 26.336 ~ 28.112/  -1.788 ~  -0.640 | 47.381 ~ 50.095/  -2.783 ~  -1.448 | 81.868 ~ 84.880/  -2.656 ~  -1.139 |

| Unit: mm | Down configuration | | | Up configuration | | |
| --- | --- | --- | --- | --- | --- | --- |
| Distance error in active driving part:  experiment - simulation (0 turn)/  experiment - simulation (1 turn) | Small  curvature | Middle  curvature | Large  curvature | Small  curvature | Middle  curvature | Large  curvature |
| $\mu$ of errors | 7.862/  2.239 | 10.413/  1,981 | 15.370/  2.163 | 6.230/  1.638 | 10.825/  2.056 | 15.858/  2.163 |
| $\sigma$ of error | 0.435/  0.325 | 0.319/  0.530 | 0.389/  0.431 | 0.299/  0.432 | 0.105/  0.488 | 0.264/  0.374 |
| Range of 1$\sigma$ for error | 7.427 ~ 8.297/  1.914 ~ 2.563 | 10.094 ~ 10.732/  1.450 ~ 2.511 | 14.981 ~ 15.759/  1.732 ~ 2.594 | 5.931 ~ 6.529/  1.206 ~ 2.070 | 10.720 ~ 10.930/  1.568 ~ 2.544 | 15.594 ~ 16.122/  1.789 ~ 2.538 |
